# The Mean Vertigo Score (MVS) Outcome Scale and Its Use in Clinical Research for Quantifying Vestibular Disorders

**DOI:** 10.3389/fneur.2021.601749

**Published:** 2021-05-05

**Authors:** Volker W. Rahlfs, Helmuth Zimmermann

**Affiliations:** idv-Data Analysis and Study Planning, Gauting, Germany

**Keywords:** mean vertigo score (MVS), multivariate analysis, Wei-Lachin procedure, composite index, power of procedures

## Abstract

**Introduction:** The Mean Vertigo Score (MVS) is a composite score for defining the burden of disease of patients suffering from vestibular disorders. It has been used in clinical research for about 30 years. This study investigates discriminant validity of the MVS and describes structural relationships of the 12 single criteria used for construction of the MVS.

**Materials and Methods:** The statistical analyses are based on the raw data of an earlier conducted randomized, doubleblind, placebo-controlled clinical trial, which compared the following four randomized treatment groups: a fixed combination of cinnarizine and dimenhydrinate (Arlevert), two groups with only one of the two study drugs, and a group with placebo. The method used for the statistical calculations is the Wei-Lachin procedure, a multivariate generalization of the Mann-Whitney test, which takes into account correlations among the 12 single symptoms of the composite score.

**Results:** All 12 single symptoms of the composite endpoint proved to be useful for detecting differences (Mann-Whitney effect size measures: 0.58–0.73) and thus for discriminating between treatment groups. Their Pearson product-moment correlations are all positive (range 0.07–0.71) and point to the same direction, which indicates one-dimensionality and good internal consistency of the composite index MVS. Furthermore, our statistical calculations revealed that successively increasing the number of single items of the MVS to up to twelve enhances its reliability (*R*_12_ = 0.923), which leads to a substantially higher test power and reduction of the number of patients needed (sample size) in a clinical trial.

**Conclusion:** The use of the multivariate Wei-Lachin procedure provides further evidence of the validity of the 12-item composite score MVS, based on the efficacy data of its 12 single vertigo symptoms. The present findings demonstrate that the MVS is a powerful tool, which can be used to adequately describe the patients' self-perceived vertigo complaints, both qualitatively and quantitatively. It may therefore be regarded as a clinically meaningful alternative to other questionnaires that are presently used in vestibular research.

## Introduction

The Mean Vertigo Score (MVS) outcome scale, which has been used as primary efficacy endpoint in several clinical studies [e.g., (1, 2)], is a composite endpoint developed for measuring the degree of vertigo in patients suffering from various vestibular disorders. It is composed of 12 single items selected in discussions with experts in the field of vestibular research. The composite outcome scale is the mean of the 12 criteria, the intensities of which are each rated by the patient on a 5-point visual analog scale ranging from 0 (no symptom) to 4 (very strong symptom), i.e., the MVS ranges from 0 to 4. Six spontaneous (unprovoked) symptoms and six vertigo symptoms in consequence of triggering factors are used for the summarizing index value:

Symptoms

- Dystasia and walking unsteadiness (DYSTAS)
- Staggering (STAGGER)
- Rotary sensation (ROTARY)
- Tendency to fall (FALL)
- Lift sensation (LIFT)
- Blackout (SCOTO)

Triggering factors

- Change of position (lying) (CHANGE)
- Bowing (BOW)
- Getting up (GETUP)
- Driving by car/train (DRIV)
- Head movements (inclination, twist) (HEADMOV)
- Eye movement (EYEMOV)

An overall good reliability of the MVS has been demonstrated previously by means of Cronbach's alpha calculations, with alpha coefficients ranging between 0.8 and 0.9 (2). Cronbach's alpha is a measure of internal consistency of a test or scale and describes the extent to which all items measure the same concept or construct; recommended alpha values range between 0.7 and 0.9 (3). Moreover, a robustness analysis showed that deletion of single items (one by one) resulted in little change of Cronbach's alpha coefficients, which indicates that there are no unnecessary duplications or redundancies among the 12 single components of the MVS.

In this paper, some new facts and characteristics of the MVS scale are reported, based on a more detailed analysis of the raw data from a previously conducted randomized, double-blind, placebo-controlled study, which compared the fixed combination of cinnarizine 20 mg and dimenhydrinate 40 mg (Arlevert) with cinnarizine 50 mg, dimenhydrinate 100 mg, and placebo (1).

We performed statistical calculations based on the efficacy raw data of the described clinical study using the Wei-Lachin procedure, a multivariate directional test, which provides information about the characteristics and validity of the MVS composite score. Taking data from this clinical trial as an example was most useful, because it has been a placebo-controlled trial. We verified the correctness and completeness of the obtained raw data by comparison with the efficacy results reported earlier. Although some additional statistical calculations were carried out, it was not the aim of our study to draw any new conclusions on drug efficacy or clinical implications beyond those already reported by Pytel et al. (1).

The following main topics will be addressed in the present paper:

1. Discriminant validity of the 12-item composite index MVS
2. Relations (correlations) between the single items of the composite index (demonstration of one-dimensionality and internal consistency of the MVS)
3. Reliability of the MVS depending on number of single items
4. General aspects concerning the use of composite endpoints in clinical trials; number of patients needed (sample size) and test power.

The clinical data used for our analysis were provided by the sponsor of the clinical trial (Hennig Arzneimittel, Germany). The results of the statistical analyses reported in (1) were based on exactly the same data records; it was the ITT data set with a total of 239 cases, with the following group sizes: Arlevert *N* = 61, Cinnarizine *N* = 61, Dimenhydrinate *N* = 59 and Placebo *N* = 58. Data were raw data for the intensities of the 12 single symptoms of the MVS, for baseline (T0), after 1 week (T1) and after 4 weeks (T2). It has to be noted that there were missing values at week 4 for a total of 7 patients, which were imputed using the procedure “Last Value Carried Forward.” More information about this data set is given in (1), and details on study design as well as demographic and clinical characteristics of the study population are briefly summarized below.

### Study Design and Treatment Regimen

In a prospective, randomized, double-blind, active- and placebo-controlled, multicenter, parallel-group clinical trial, the efficacy and safety of a fixed combination of cinnarizine 20 mg and dimenhydrinate 40 mg (Arlevert) was compared with cinnarizine 50 mg, dimenhydrinate 100 mg, and placebo, each given three times daily for 4 weeks to patients with vestibular vertigo.

### Study Population

Male and female outpatients, aged above 30 years, with vertigo of central, peripheral or combined central/peripheral vestibular vertigo were eligible for enrollment into the clinical trial. Patients had to rate at least one of six vertigo symptoms with at least “medium” intensity (i.e., score of ≥2 on a 5-point VAS ranging from 0 to 4) and show abnormal vestibulospinal movement patterns, which were registered by means of craniocorpography while performing Romberg and Unterberger Tests. None of the study participants underwent any physical or occupational rehabilitation therapy during the 4-week treatment phase.

A total of 246 patients were included in the clinical trial; the mean age was 51.2 years and about two thirds were female (63.8%). The four treatment arms were equally balanced with respect to demographic data (age, gender, weight, height, BMI) and clinical characteristics such as duration of vertigo, number of patients with pretreatment of vertigo, concomitant diseases, and concomitant medications. Of the randomized patients, 38 (15.4%) suffered from peripheral vertigo, 49 (19.9%) from central, and 159 (64.7%) from combined central/peripheral vertigo, each similarly distributed among the 4 treatment groups. A total of 18 discrete underlying diseases were identified (ICD-10 codes), leading either to peripheral (e.g., vestibular neuropathy, labyrinthitis, labyrinth contusion) or central vertigo (e.g., vertebrobasilar ischemia, vascular encephalopathy, basilar impression, cerebral contusion). Patients had suffered from vertigo on average for 2.6 years, and ~40% of patients in each group had taken antivertigo drugs, such as betahistine, pentoxyphylline or cinnarizine (38, 25, and 12 patients, respectively), before enrollment into the study. The majority (57–69%) of patients had concomitant diseases, e.g., cardiovascular diseases (~40% of patients) or disorders of the locomotor apparatus (~20%); accordingly, about 40% of concomitant medications were cardiovascular drugs, around 13% were drugs acting on the central nervous system, and about 9% were analgesics and antirheumatics.

### Validation of Data Transfer

The raw data for each of the 239 patients with complete data were delivered as an Excel table; transfer of the table to our validated statistics program TESTIMATE was performed in a validated environment. Thus, we calculated for each point in time (T0, T1, T2) the MVS for each group: mean, standard deviation, min, max, and number of cases. There was nearly perfect agreement with the values in the sponsor's Clinical Study Report and in (1); there were only rounding differences in the third significant digit. The same consistency was confirmed for the change from baseline values. The study report and the publication (1) reported also Wilcoxon-Mann-Whitney estimators and their confidence intervals for the comparisons of Arlevert and the three other groups. We re-calculated these values with our program and obtained perfect agreement with the effect size measures; however, some small insignificant differences were found for the confidence intervals, which may be attributable to slightly different formulas for calculation of these values cited in the statistical literature. All in all we could confirm that the delivered data are verifiably identical to the original data set.

The Program TESTIMATE which was used for our calculations is a proprietary program and is available upon request by addressing the corresponding author.

## Statistical Methods

The present analysis was performed using the Wei-Lachin procedure, a multivariate generalization of the Wilcoxon-Mann-Whitney test, which takes account of the correlations among univariate Mann-Whitney tests for each outcome to produce an overall average estimate of benefit. The summarizing test is a directional test that is most efficient in case of known direction of superiority (4). The procedure is described by Wei and Lachin (5) and Lachin (6). Practical examples are given in modern textbooks on multiple testing problems [see e.g., (7)].

The analysis delivers the following results:

1. Mann-Whitney (MW) effect size measures, which represent the probability that a randomly chosen subject of the test group (Y) is better off than a randomly chosen subject of the comparison group (X), with probability ranging from 0 to 1, and 0.5 indicating equality; it is statistically defined as *P* (X < Y) + 0.5 *P* (X = Y). The relevant benchmark values for the MW effect size measure may be defined as follows (8, 9): 0.29 (large inferiority), 0.36 (medium inferiority), 0.44 (small inferiority), 0.50 (equality), 0.56 (small superiority), 0.64 (medium superiority), and 0.71 (large superiority).
2. Intercorrelations between single components of the MVS (Pearson product-moment correlations).
3. For group comparisons: *P*-values and MW effect size measures for each single symptom and for the pooling procedure average effect.

**Figure 1:**
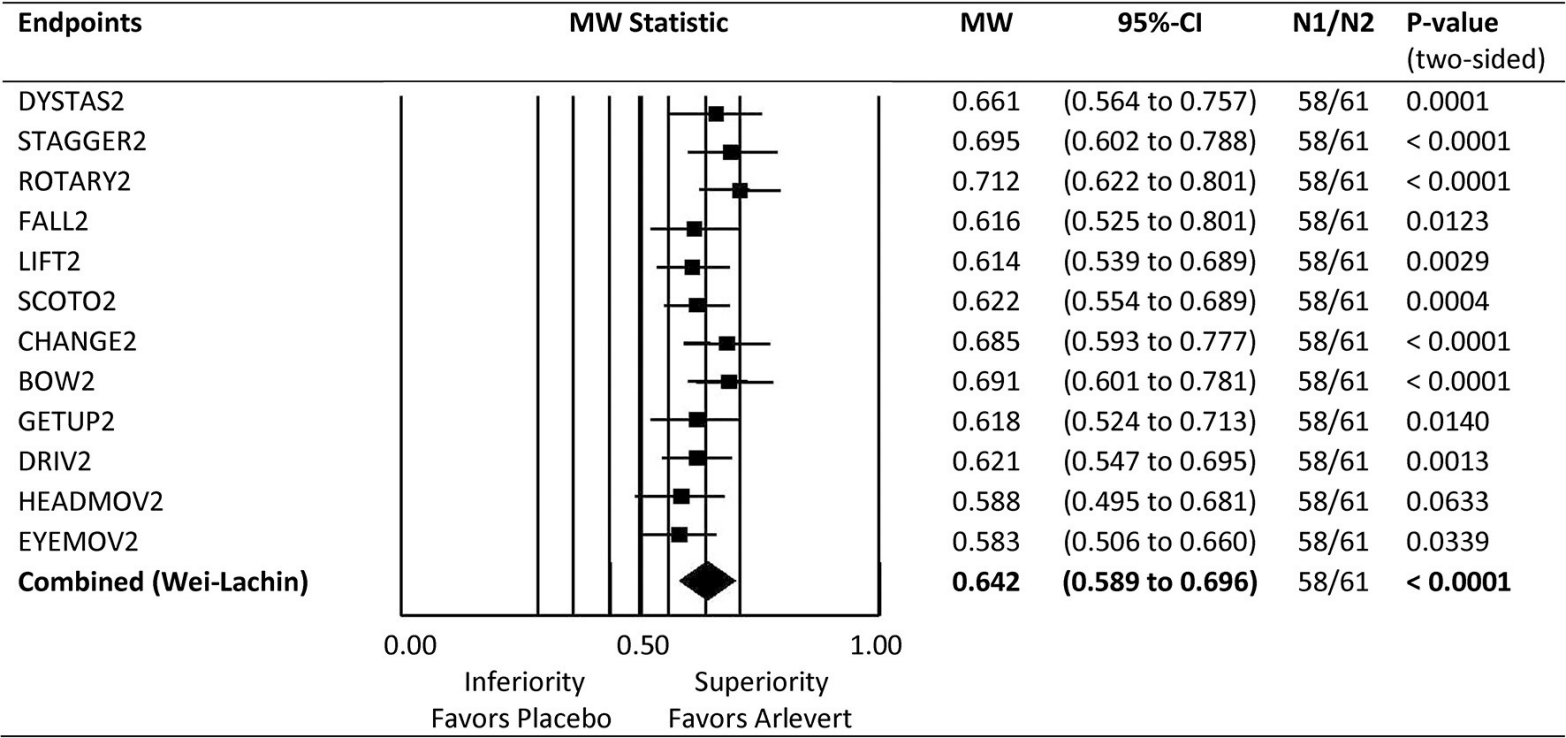
Forest plot of Mann-Whitney (MW) effect size measures for comparison of Arlevert with Placebo after 4 weeks of treatment. Results are given for single endpoints and combined (composite) endpoint. MW: MW effect size measures; Wilcoxon test for single endpoints, directional Wei-Lachin test for combined endpoint. 95%-CI: lower and upper bounds of 95% confidence intervals. N1, N2: number of patients treated with Placebo and Arlevert, respectively.

Further statistical procedures applied in this paper:

- Forest plot graph to show all 12 MW-values and their confidence intervals in juxtaposition to describe dimensionality of the MVS composite index (Figure 1).
- Definition of reliability (Appendix) and definition of the reliability of a composite index based on the Spearman-Brown formula for pooling several items (10, 11).
- Calculation of sample size in a clinical study when applying the Wei-Lachin pooling procedure and the concept of reliability associated with a simple composite index, both taking into account the number of items and their (average) correlations (**Table 3**).

When comparing the multivariate approach applying the Wei-Lachin procedure with the concept based on a composite endpoint, an interesting feature is efficiency, the number of patients necessary when performing a clinical study. The efficiency of the Wei-Lachin procedure can be quantified with the formula

$$\begin{array}{l} n_{m}/n_{1}:=\left[\left(m-1\right)\varrho +1\right]/m \end{array}$$

where m is the number of criteria, ρ is the average of the correlation coefficients and *n*_*m*_/*n*_1_ is the sample size of all criteria used, divided by the sample size for only one criterion (12–14).

Another formula makes use of the well-known concept of reliability using the Spearman-Brown formula for pooling several components (10, 11, 15). The formula for obtaining reliability, based on several components, is:

$$\begin{array}{l} R_{m}=m\cdot R/\left[\left(m-1\right)\cdot R+1\right] \end{array}$$

with *R* being the reliability of a single component, *R*_*m*_ the reliability of m components combined (16).

## Results

There were 6 two-group comparisons available with 4 treatment groups. All two-group comparisons were analyzed for 3 points in time, namely at baseline, after 1 week, and after 4 weeks of treatment; thus, there were 18 analysis calculations.

A condensed form of the most important results of our multivariate analysis is given in Table 1. The table gives information on all 18 analyses: Mann-Whitney (MW) effect size measures and *P*-values, for 3 points in time: T0 (before treatment), T1 (after 1 week), and T2 (after 4 weeks). MW values are given in the upper right of the table in matrix form, whereas *P*-values are provided in the lower left. It is interesting that only the comparisons of Arlevert with Cinnarizine, Dimenhydrinate and Placebo after 4 weeks of treatment are statistically significant with impressive values *P* < 0.001; the corresponding MW values are 0.605, 0.615, and 0.642. The value 0.642 indicates a relevant difference, whereas the other two values are located between a small (0.56) and a medium difference (0.64). It is noteworthy that the difference between Cinnarizine and Placebo as well as that between Dimenhydrinate and Placebo is not statistically significant (*P* = 0.184 and *P* = 0.348, respectively). Thus, the fixed combination Arlevert is clearly superior to each of the two monodrug preparations and needs each of the two single active components for producing a benefit as compared to placebo.

**Table 1:** Selected results of multivariate data analysis.

		**Arlevert**	**Cinnarizine**	**Dimenhydrinate**	**Placebo**
Arlevert	T0	-------------	0.467	0.460	0.471
	T1		0.536	0.547	0.572
	T2		0.605	0.615	0.642
Cinnarizine	T0	0.126	-------------	0.494	0.506
	T1	0.175		0.508	0.535
	T2	<0.001		0.511	0.539
Dimenhydrinate	T0	0.086	0.785	-------------	0.511
	T1	0.068	0.748		0.526
	T2	<0.001	0.670		0.528
Placebo	T0	0.235	0.812	0.668	-------------
	T1	0.011	0.218	0.339	
	T2	<0.001	0.184	0.348	

*The table presents some results of the multivariate analysis in condensed form, Mann-Whitney effect size measures and P-values for the comparisons of treatment groups for each of 3 points in time, T0 (before treatment), T1 (after 1 week), T2 (after 4 weeks). MW measures are in the upper right half of the table; P-values are in the lower left half of the table*.

Comparison of Arlevert and Placebo at Week 4 is standing out here and will therefore be considered in more detail. Figure 1 gives a forest plot overview for the results of the 12 single outcome variables as well as for the combination value. Mann-Whitney (MW) effect size values, their 95%-confidence intervals and *P*-values are given on the right side of the figure. The combined MW-value is just an average of the 12 single MW values. It is of interest to see that in principle all 12 criteria are useful for detecting differences between groups (MW values range between 0.58 and 0.71), only “head movements” and “eye movements” being slightly less important (MW values of 0.588 and 0.583, respectively). Of the 12 criteria (symptoms), a large superiority of the combination drug (MW ≥ 0.71) was observed for ROTARY2, 4 criteria showed a medium superiority (MW ≥ 0.64; DYSTAS2, STAGGER2, CHANGE2, BOW2), and the remaining criteria a small superiority (MW ≥ 0.56). Thus, all 12 criteria are useful for discriminating between treatment groups. Furthermore, the summarizing combined value has a very small confidence interval, resulting from making use of all correlations between the 12 criteria. Both features demonstrate a good discriminant validity of the composite endpoint MVS. In addition, Figure 1 shows that all 12 components point to the same direction, with non-relevant differences between Mann-Whitney effect size measures, a *desideratum* of a good composite score according to Sankoh et al. (17).

Table 2 shows the correlation matrix of the 12 single MVS items for comparison of the fixed-combination product with placebo after 4-week treatment. Correlation analysis provides information on the strength and direction of a relationship between variables; correlation coefficients are dimensionless and range between −1 and +1. The theory of the correlation-sensitive Wei-Lachin procedure maintains that criteria with higher correlation to others contribute less to the overall combined result. For example “Dystasia/walking unsteadiness” and “Staggering” have a correlation of *r* = 0.72, not exceptional and thus easily included in the self adjusting process of the Wei-Lachin procedure, whereas the two variables “Dystasia/walking unsteadiness” and “Tendency to fall” are rather moderately correlated (*r* = 0.54). All single components show positive correlations, which is an indication for the one-dimensionality and good internal consistency of the combined endpoint MVS.

**Table 2:** Correlation matrix for the 12 single MVS criteria (Pearson product-moment correlations) after 4 weeks of treatment.

**Criterion**	**DYSTAS2**	**STAGGER2**	**ROTARY2**	**FALL2**	**LIFT2**	**SCOTO2**	**CHANGE2**	**BOW2**	**GETUP2**	**DRIV2**	**HEADMOV2**	**EYEMOV2**
DYSTAS2	1.00	0.72	0.48	0.54	0.21	0.23	0.61	0.51	0.48	0.18	0.49	0.13
STAGGER2		1.00	0.52	0.64	0.25	0.18	0.57	0.48	0.40	0.12	0.62	0.14
ROTARY2			1.00	0.35	0.26	0.21	0.47	0.34	0.35	0.06	0.28	0.23
FALL2				1.00	0.29	0.17	0.45	0.38	0.32	0.02	0.35	0.16
LIFT2					1.00	0.24	0.21	0.21	0.23	0.16	0.14	0.26
SCOTO2						1.00	0.21	0.11	0.19	0.08	0.01	0.07
CHANGE2							1.00	0.64	0.59	0.14	0.52	0.10
BOW2								1.00	0.74	0.09	0.52	0.21
GETUP2									1.00	0.16	0.45	0.25
DRIV2										1.00	0.20	0.13
HEADMOV2											1.00	0.32
EYEMOV2												1.00

In the following we describe some quantitative measures and their relationships with respect to the efficiency of a multi-component composite index. The clinical study, the data of which were used for our statistical calculations, would be clearly undersized for a single variable: Sample sizes for just one confirmatory test would have required about 90 patients per group (relevant difference MW = 0.64, α = 0.05, β = 0.20). Nevertheless, some interesting results were obtained using the Wei-Lachin procedure for the analysis. In principle the procedure gives a summarizing composite index, which is very powerful because the correlations between single endpoints are included in the model. Of interest, endpoints with higher correlations to other endpoints contribute less than those with lower correlations. Thus, the information content of the single components is automatically adjusted for, and the result for the composite index is obtained with high precision.

Table 3 gives an overview of the overall precision of a study depending on the number of replications (components 1–12) used in a clinical trial. Please note that we have assumed the reliability of one component as *R* = 0.5, a value generally regarded as low. As can be seen, enlarging the number of components both reduces the number of patients needed and enlarges the reliability. The number of patients needed can be inferred depending upon reliability. The number is defined as *n*_1_·*R*_1_/*R*_*m*_ with *R*_*m*_ as reliability for an assumed number of components. Just 3 components would suffice to reduce the number of patients per group from 90 to 60 (see numbers in boldface), about the number that has been included in the clinical trial of Pytel et al. (1). With this configuration the reliability is raised from 0.5 to 0.750, which means that a composite score with just 3 components would be a reasonable outcome measure for allowing the study to be performed with 60 patients. It is noteworthy that the 12-components MVS is considerably improved as can be inferred from the calculated numbers in the table. The reliability goes up to *R*_12_ = 0.923 and the sample size of a clinical trial would be acceptable by including only 49 patients, based on the same alpha and beta level as well as the same difference regarded as important. Thus, the 12-component MVS enhances the test power in clinical studies substantially, but is also very useful for diagnosing single patients where high reliability is required. All formulas used in this paragraph are given in the Appendix.

**Table 3:** Precision of a study depending on the number of replications of measurement; for the value ϱ = *R* = 0.5, sample size according to the Wei-Lachin procedure, and reliability.

***n_***m***_* number of cases by *m* replications**	***m* number of replications**	***n_***m***_*_**/**_*n*_**1**_ Wei-Lachin**	***R_***m***_* reliability by *m* replications**
90	1	1.000	0.500
68	2	0.750	0.667
**60**	**3**	0.667	0.750
56	4	0.625	0.800
54	5	0.600	0.833
53	6	0.583	0.857
51	7	0.571	0.875
51	8	0.563	0.889
50	9	0.556	0.900
50	10	0.550	0.909
49	11	0.545	0.917
49	12	0.542	0.923

*The product Wei-Lachin by reliability is constant = 0.5*.

Thus, it is in principle advantageous to use several components either summarized by a composite index or by the Wei-Lachin procedure. A composite index, if based on well-known components, is a powerful instrument in clinical research, because either a smaller number of experimental units (patients) could be used (reduced sample size) or else a smaller difference could be detected because of a substantial enhancement of the test power of the clincial trial.

## Discussion

Vertigo is a multifaceted symptom complex resulting from a variety of possible underlying vestibular disorders. Registration of the patient's self-reported subjective perception of vertigo is generally regarded as the most useful instrument to establish medical history and control of therapeutic success. For this purpose, various vertigo-related questionnaires (patient-reported outcomes) have been developed, among others the Dizziness Handicap Inventory [DHI (18)], Vertigo Symptom Scale [VSS (19)], and Vertigo, Dizziness, Imbalance [VDI (20)] questionnaire [for a review see (21)]. These questionnaires are composed of a relatively large number of items, which include, in addition to vertigo-specific symptoms, a considerable number of further items, such as concomitant symptoms frequently associated with vertigo (e.g., nausea, vomiting) as well as items related to a symptomatology that adversely affects the patient's daily activities and thus health-related quality of life caused by vestibular disorders.

In the present paper, we investigated a composite vertigo index named Mean Vertigo Score (MVS), the components of which had been selected to reflect the patient's subjective self-reported cardinal vertigo symptoms and thus, as opposed to the above-mentioned instruments, is a “vertigo symptom-related” questionnaire. After the MVS had been developed in co-operation with various experts in the field of vestibular research, it has been used as primary efficacy endpoint in a number of clinical trials [e.g., (1, 2)] as patient-reported outcome to evaluate therapeutic success of medical treatment. Item-selection of the MVS was mainly based on the involved clinical expert's experience from their daily medical practice. As the questionnaire is filled out by the patients themselves, the terms were kept as simple as possible and in everyday language to be easily comprehensable to the average vertigo patient.

The 12-item composite index MVS contains 6 spontaneous (unprovoked) vertigo symptoms and 6 symptoms provoked by specific body movements (i.e., vertigo triggering factors). Some symptoms, such as “rotary sensation” and “tendency to fall” (i.e., lateral pulsion directed to the affected side) are typical for peripheral vestibular vertigo, whereas others, such as “dystasia and walking unsteadiness” or “staggering” (generally undirected) are more typical for central vestibular disorders. The concept of distinguishing between “spontaneous” and “triggered” vertigo symptoms is largely in line with a consensus document of the “Committee for the Classification of Vestibular Disorders” of the Bárány Society, published by Bisdorff et al. (22). The authors attempted to harmonize the terminology of vestibular symptoms, involving contemporary internationally recognized neurologists and otolaryngologists from various countries, with the aim to “guide investigators conducting clinically-oriented vestibular research.” Furthermore, it has been the declared aim of the consensus paper to establish a classification of vestibular symptoms that are non-hierarchical and non-overlapping, a condition that is also largely met by the composition of the MVS.

A good reliability and internal consistency of the composite endpoint has been demonstrated earlier by means of Cronbach's alpha calculations (2). The primary objective of the present paper was to further characterize and validate the MVS.

Figure 1 gives an overview of all Mann-Whitney (MW) effect size measures for the single endpoints (symptoms) and the summary (combined) value. All single endpoints give substantial evidence for superiority of the fixed-combination drug and proved to be useful for detecting differences and thus for discriminating between treatment groups. Furthermore, the MW effect size values are very similar and tend in the same direction, which is a prerequisite for a good composite endpoint (17). The MW values range between 0.58 and 0.71, so there is no one single endpoint really weak for discrimination between treatment groups. Thus, the ensemble of vertigo symptoms for construction of the composite index seems to be well-chosen, leading to a good discriminant validity of the MVS.

Another interesting feature of our results is the correlation matrix for all single criteria (Table 2). Inspection of the matrix shows that correlations between the single criteria are all positive, and they all point to the same direction, which is a general prerequisite when choosing single components for construction of a composite endpoint; furthermore, this indicates one-dimensionality and good internal consistency of the 12-item composite index MVS. The relatively large range of correlation values (0.07–0.72) is an indication of the generally multifaceted nature of the disease and presumably may also be attributed to the largely heterogeneous patient population of the clinical trial, which included patients suffering from central, peripheral or combined central-peripheral vertigo.

Construction of a composite endpoint is based on ethical and economic considerations: the higher the sensitivity of a composite endpoint, the fewer the number of subjects needed to achieve sufficient power to detect a meaningful treatment effect in a clinical trial. Moreover, there is no multiplicity issue if the trial has a single composite endpoint and a single global claim as its objective (14). Our statistical calculations revealed that augmenting the number of single items of the MVS up to twelve substantially increased its reliability, which enhances the test power and reduces the necessary sample size of a clinical trial. This also explains the high discriminant validity of the 12-item composite index MVS.

The present paper provides evidence for the usefulness of the composite index MVS as an instrument for recording the patient's subjective (self-assessed) vertigo symptoms. In a first step, various promising aspects regarding reliability and validity of the MVS are reported, but additional work is needed to provide further evidence for its role as a clinically meaningful patient-reported outcome measure in vestibular research. While our findings demonstrate a good reliability and internal consistency as well as one-dimensionality and good discriminant validity of the MVS, further features of validation still remain to be established. Although the single symptoms of the composite index were selected in close co-operation with experienced neurotologists, patients suffering from vertigo were not directly involved in item-selection of the MVS. Furthermore, correlation of the patients' self-assessed (subjective) symptoms with an accepted “gold standard,” such as any objective measurements (e.g., post-urography) used in vestibular research, is yet to be examined, even though generally rather weak correlations may be expected mainly due to the complexity of the disease. Finally, our results reported in this paper are based on the data of just a single clinical trial, with a rather small and quite heterogeneous patient population. Therefore, generalizability of our findings to other patient populations needs to be investigated in future research.

## Conclusion

In the present paper, we provide further evidence for the validity of the 12-item composite index MVS with respect to its potential use in vestibular research. Based on the efficacy raw data taken from a clinical trial previously conducted and reported by Pytel et al. (1), we applied the multivariate Wei-Lachin procedure as a tool to further characterize the MVS, which was used as primary efficacy endpoint in the respective clinical trial, and the structured characteristics of the selection of its 12 single components (6 spontaneous and 6 triggered vertigo symptoms).

Overall, our findings demonstrate a good discriminant validity, one-dimensionality as well as good internal consistency and reliability of the MVS.

In conclusion, the 12-item MVS is believed to be a powerful tool and clinically meaningful instrument for registration and evaluation of self-reported symptoms of patients suffering from various kinds of vertigo, both in clinical research for comparison of therapeutic success in different treatment groups and as a supporting measure to improve establishment of medical history and diagnosis in individual patients in daily clinical routine. As the single items of the composite index are restricted to cardinal vertigo symptoms, the MVS may be a useful addition or alternative option to other composite scores presently applied in vestibular research.

## Data Availability Statement

All datasets generated or analyzed for this study are included in the manuscript/Supplementary files.

## Ethics Statement

The original study was reviewed and approved by the Ethics Committees of the participating study sites. The patients/participants provided their written informed consent to participate in this study.

## Author Contributions

VR: conceived and designed the methodological research, analyzed the data, and wrote the paper. HZ: designed and checked mathematical part. All authors contributed to the article and approved the submitted version.

## Conflict of Interest

The firm Hennig gave honoraria for re-analysis of data and writing the report.

## Appendix

### Formulas for Reliability and Number of Cases

**Table:**

**Reliability**	**Number of cases**
(1) $R=\frac{\sigma_{T}^{2}}{\sigma_{T}^{2}+\sigma_{e}^{2}}$	(2) $n^{*}=2\;{\left(\frac{\sigma_{T}}{\vartheta }\right)}^{2}\cdot {\left(z_{\alpha /2}+z_{\beta }\right)}^{2}$
**With error:** ***m*** **repeated measurements per cases and** ***n*** **cases per group**	
(3) $R_{m}=\frac{\sigma_{T}^{2}}{\sigma_{T}^{2}+\sigma_{e}^{2}/m}=\frac{m\cdot R}{\left(m-1\right)R+1}$ $\frac{\sigma_{e}^{2}}{\sigma_{T}^{2}}=m\;\cdot \left(1/R_{m}-1\right)$	(4) $n_{m}=\frac{n^{*}}{R_{m}}\;$= $\frac{2}{R_{m}}{\left(\frac{\sigma_{T}}{\vartheta }\right)}^{2}\cdot {\left(z_{\frac{\alpha }{2}}+z_{\beta }\;\right)}^{2}$ $=\;\frac{2\left(\sigma_{T}^{2}+\sigma_{e}^{2}/m\right)\cdot {\left(z_{\alpha /2}+z_{\beta }\right)}^{2}}{\vartheta^{2}}\;$
(5) $n_{m}\cdot R_{m}=n^{*}$	

(1) Formula for reliability, defined as a function of treatment variance $\sigma_{T}^{2}$ and error variance $\sigma_{e}^{2}$.

(2) Formula for sample size calculating using critical values *z*_α/2_ and *z*_β_ with $\frac{\sigma_{T}}{\vartheta }$ the difference to be detected.

(3) Formula for reliability, defined as a function of treatment variance $\sigma_{T}^{2}$ and error variance modified by the number m of replications sigma $\sigma_{e}^{2}/m$.

(4) Number of cases needed when m repeated measurements are given.

(5) Number of cases multiplied with reliability is constant (number of cases when no error).
