# Supplementary material for: The Mean Vertigo Score (MVS) Outcome Scale and Its Use in Clinical Research for Quantifying Vestibular Disorders
Source: Front Neurol. 2021 May 5;12:601749. doi: 10.3389/fneur.2021.601749 (PMC8131667; doi:10.3389/fneur.2021.601749)
Supplement: Supplementary file 1 [file Table_1.pdf]

Supplementary Table 1a: Intensities of 6 spontaneous vertigo symptoms in the course of 4 weeks of treatment

PATNO	MEDICAT	DYSTAS0	DYSTAS1	DYSTAS2	STAGGER0	STAGGER1	STAGGER2	ROTARY0	ROTARY1	ROTARY2	FALL0	FALL1	FALL2	LIFT0	LIFT1	LIFT2	SCOTO0	SCOTO1	SCOTO2
1	CIN	3.0	2.0	1.0	3.0	2.0	2.0	2.0	1.0	0.0	1.0	0.0	0.0	1.0	0.0	0.0	0.0	0.0	0.0
2	CIN	4.0	3.0	2.0	3.0	2.0	2.0	3.0	2.0	1.0	2.0	2.0	1.0	2.0	1.0	0.0	2.0	1.0	0.0
3	CIN	1.5	1.0	0.0	2.0	2.0	1.0	2.0	1.0	1.0	2.0	1.0	1.0	0.0	0.0	0.0	1.0	1.0	0.0
4	PLAC	1.5	1.0	1.0	2.0	1.0	1.0	2.5	0.0	0.0	2.0	1.0	1.0	1.0	1.0	1.0	2.0	0.0	1.0
5	DIM	2.0	2.1	2.0	2.0	2.0	1.1	2.0	2.0	0.0	0.0	0.0	0.0	0.0	0.0	0.0	0.0	0.0	0.0
6	DIM	2.0	1.0	0.0	2.0	2.0	1.0	3.0	2.0	2.0	0.0	0.0	0.0	0.0	0.0	0.0	0.0	0.0	0.0
7	DIM	2.0	1.0	1.0	1.0	1.0	0.0	0.0	0.0	0.0	0.0	0.0	0.0	0.0	0.0	0.0	0.0	0.0	1.0
8	PLAC	2.0	2.0	1.0	0.0	0.0	0.0	0.0	0.0	0.0	0.0	0.0	0.0	0.0	0.0	0.0	1.0	1.0	1.0
9	PLAC	4.0	1.0	0.0	1.0	1.0	0.0	4.0	0.0	0.0	0.0	0.0	0.0	0.0	0.0	0.0	3.0	0.0	0.0
10	DIM	1.0	1.0	2.0	0.0	0.0	0.0	2.0	2.0	2.0	2.0	2.0	2.0	0.0	0.0	0.0	2.5	0.0	0.0
11	ARL	2.0	0.0	0.0	0.0	0.0	0.0	4.0	0.0	0.0	0.0	0.0	0.0	0.0	0.0	0.0	0.0	0.0	0.0
12	CIN	4.0	4.0	2.0	4.0	4.0	2.0	3.0	3.0	3.0	0.0	0.0	0.0	0.0	0.0	0.0	0.0	0.0	0.0
13	PLAC	3.0	2.0	1.0	2.0	2.0	2.0	3.0	2.0	1.0	2.0	2.0	2.0	0.0	0.0	0.0	0.0	0.0	0.0
14	PLAC	2.0	1.0	0.0	1.0	0.0	0.0	2.5	1.0	1.0	0.0	0.0	0.0	0.0	0.0	0.0	0.0	0.0	0.0
15	DIM	3.0	2.0	2.0	3.0	2.0	2.0	2.0	2.0	1.0	2.0	1.0	1.0	1.0	1.0	1.0	0.0	0.0	0.0
16	PLAC	3.0	3.0	2.0	3.0	3.0	2.0	4.0	3.0	2.0	2.0	2.0	2.0	0.0	0.0	0.0	0.0	0.0	0.0
17	ARL	4.0	3.0	2.0	3.0	2.0	0.0	1.0	0.0	0.0	3.0	2.0	0.0	3.0	2.0	0.0	3.0	2.0	0.0
18	CIN	3.0	3.0	2.0	3.0	3.0	3.0	2.0	1.0	1.0	1.0	1.0	0.0	0.0	0.0	0.0	1.0	1.0	0.0
19	CIN	3.0	2.0	1.0	3.0	2.0	1.0	1.0	1.0	1.0	1.0	1.0	0.0	0.0	0.0	0.0	0.0	0.0	0.0
20	PLAC	3.0	2.0	2.0	3.0	2.0	1.0	4.0	2.0	2.0	2.0	2.0	0.0	2.0	1.0	0.0	1.0	0.0	0.0
21	CIN	2.0	1.0	0.0	0.0	0.0	0.0	0.0	0.0	0.0	0.0	0.0	0.0	0.0	0.0	0.0	0.0	0.0	0.0
22	CIN	2.0	1.0	0.0	1.0	1.0	0.0	2.0	0.0	0.0	2.0	0.0	0.0	0.0	0.0	0.0	1.0	0.0	0.0
23	PLAC	3.0	2.0	1.0	3.0	1.0	1.0	2.0	2.0	1.0	2.0	1.0	0.0	1.0	1.0	0.0	0.0	0.0	0.0
24	CIN	2.0	1.0	0.0	0.0	0.0	0.0	3.0	0.0	0.0	2.0	1.0	1.0	2.0	2.0	0.0	0.0	0.0	0.0
25	PLAC	3.0	2.0	2.0	3.0	2.0	1.0	1.0	1.0	1.0	0.0	0.0	0.0	0.0	0.0	0.0	0.0	0.0	0.0
26	PLAC	4.0	3.0	1.0	3.0	2.0	1.0	3.0	2.0	0.0	3.0	1.0	0.0	2.0	1.0	0.0	2.0	1.0	0.0
27	DIM	2.0	1.0	1.0	1.0	0.0	0.0	4.0	3.0	1.0	0.0	0.0	0.0	0.0	0.0	0.0	1.0	0.0	0.0
28	DIM	3.0	2.0	1.0	3.0	2.0	1.0	4.0	2.0	1.0	2.0	1.0	1.0	2.0	1.0	1.0	2.0	1.0	0.0
29	PLAC	3.0	3.0	2.0	3.0	2.0	2.0	2.0	2.0	1.0	3.0	2.0	2.0	1.0	1.0	1.0	1.0	0.0	0.0
30	CIN	2.0	2.0	2.0	2.0	2.0	2.0	4.0	3.0	3.0	2.0	2.0	2.0	2.0	2.0	2.0	1.0	1.0	1.0
31	ARL	2.0	1.0	0.0	2.0	1.0	0.0	4.0	2.0	0.0	1.0	1.0	0.0	1.0	1.0	1.0	0.0	0.0	0.0
32	DIM	2.0	1.0	1.0	2.0	1.0	1.0	0.0	0.0	0.0	1.0	1.0	1.0	0.0	0.0	0.0	0.0	0.0	0.0
33	PLAC	0.0	0.0	0.0	2.0	2.0	1.0	0.0	0.0	0.0	0.0	0.0	0.0	0.0	0.0	0.0	0.0	0.0	0.0
34	DIM	2.0	1.0	0.0	2.0	1.0	0.0	4.0	3.0	2.0	1.0	0.0	0.0	0.0	0.0	0.0	0.0	0.0	0.0
35	CIN	2.0	2.0	1.0	2.0	2.0	1.0	3.0	2.0	1.0	2.0	1.0	1.0	2.0	2.0	1.0	1.0	0.0	0.0
36	ARL	3.0	2.0	0.0	3.0	2.0	0.0	1.0	1.0	1.0	2.0	1.0	0.0	2.0	1.0	0.0	0.0	0.0	0.0
37	DIM	0.0	0.0	0.0	0.0	0.0	0.0	1.0	1.0	1.0	0.0	0.0	0.0	3.0	3.0	3.0	0.0	0.0	0.0
38	CIN	3.0	3.0	2.0	3.0	2.0	2.0	4.0	3.0	2.0	1.0	0.0	0.0	1.0	1.0	0.0	0.0	0.0	0.0
39	ARL	2.0	1.0	0.0	2.0	1.0	0.0	4.0	2.0	0.0	3.0	2.0	1.0	2.0	1.0	0.0	0.0	0.0	0.0
40	DIM	1.0	3.0	1.0	1.0	3.0	2.0	0.0	1.0	0.0	2.0	2.0	2.0	0.0	0.0	0.0	1.0	0.0	0.0
41	DIM	2.0	2.0	1.0	2.0	1.0	1.0	4.0	2.0	1.0	1.0	0.0	0.0	0.0	0.0	0.0	0.0	0.0	0.0
42	CIN	3.0	2.0	1.0	2.0	2.0	1.0	4.0	2.0	1.0	3.0	2.0	2.0	0.0	0.0	0.0	0.0	0.0	0.0
43	CIN	3.0	2.0	1.0	3.0	2.0	2.0	1.0	0.0	0.0	3.0	2.0	1.0	2.0	1.0	1.0	3.0	2.0	1.0
44	DIM	3.0	2.0	2.0	3.0	2.0	2.0	1.0	0.0	0.0	2.0	1.0	0.0	2.0	1.0	0.0	2.0	1.0	1.0
45	ARL	3.0	2.0	1.0	2.0	1.0	0.0	2.0	1.0	0.0	0.0	0.0	0.0	3.0	2.0	1.0	0.0	0.0	0.0
46	CIN	3.0	3.0	2.0	2.0	2.0	1.0	2.0	2.0	1.0	1.0	0.0	0.0	1.0	0.0	0.0	0.0	0.0	0.0
47	DIM	2.0	2.0	1.0	2.0	2.0	1.0	4.0	3.0	2.0	2.0	1.0	1.0	1.0	1.0	0.0	1.0	0.0	0.0
48	ARL	4.0	3.0	1.0	4.0	3.0	1.0	2.0	2.0	1.0	2.0	1.0	1.0	1.0	0.0	0.0	2.0	1.0	0.0
49	CIN	3.0	2.0	2.0	3.0	2.0	1.0	2.0	1.0	1.0	3.0	2.0	1.0	3.0	2.0	1.0	3.0	3.0	2.0
50	ARL	2.0	1.0	1.0	2.0	1.0	0.0	3.0	2.0	1.0	1.0	1.0	0.0	1.0	0.0	0.0	0.0	0.0	0.0
51	DIM	3.0	2.0	1.0	3.0	2.0	1.0	2.0	2.0	1.0	3.0	2.0	0.0	3.0	1.0	0.0	1.0	0.0	0.0
52	ARL	4.0	3.0	1.0	3.0	2.0	1.0	2.0	2.0	2.0	2.0	1.0	0.0	1.0	0.0	0.0	3.0	2.0	1.0
53	ARL	3.0	1.0	0.0	3.0	1.0	0.0	3.0	2.0	0.0	2.0	1.0	0.0	1.0	1.0	0.0	3.0	1.0	0.0
54	ARL	3.0	3.0	2.0	3.0	3.0	2.0	4.0	3.0	3.0	3.0	2.0	2.0	1.0	1.0	1.0	0.0	0.0	0.0
55	CIN	2.0	1.0	1.0	2.0	1.0	1.0	4.0	0.0	0.0	0.0	0.0	0.0	0.0	0.0	0.0	0.0	0.0	0.0
56	DIM	2.0	2.0	1.0	1.0	1.0	0.0	4.0	3.0	2.0	1.0	0.0	0.0	1.0	0.0	0.0	2.0	1.0	0.0
57	DIM	3.0	2.0	1.0	3.0	3.0	2.0	2.0	2.0	2.0	3.0	2.0	1.0	1.0	1.0	1.0	2.0	1.0	0.0
58	PLAC	2.0	2.0	1.0	2.0	2.0	1.0	4.0	3.0	2.0	2.0	1.0	1.0	1.0	1.0	0.0	0.0	0.0	0.0
59	PLAC	3.0	3.0	2.0	3.0	3.0	1.0	2.0	2.0	1.0	2.0	1.0	0.0	1.0	1.0	0.0	3.0	2.0	1.0
60	ARL	3.0	3.0	2.0	3.0	2.0	1.0	3.0	2.0	2.0	2.0	1.0	1.0	2.0	1.0	1.0	4.0	3.0	2.0
61	PLAC	1.0	1.0	0.0	1.0	0.0	0.0	4.0	3.0	1.0	3.0	2.0	0.0	1.0	0.0	0.0	0.0	0.0	0.0
62	ARL	3.0	1.0	0.0	3.0	1.0	0.0	2.0	1.0	0.0	4.0	2.0	0.0	3.0	2.0	1.0	3.0	2.0	0.0
63	PLAC	3.0	3.0	2.0	3.0	3.0	2.0	3.0	2.0	2.0	2.0	2.0	1.0	2.0	1.0	1.0	3.0	1.0	0.0

64	DIM	2.0	2.0	1.0	2.0	2.0	1.0	2.0	1.0	1.0	2.0	1.0	0.0	0.0	0.0	0.0	0.0	0.0	0.0
65	CIN	3.0	2.0	1.0	3.0	2.0	1.0	2.0	2.0	1.0	3.0	2.0	1.0	2.0	1.0	1.0	2.0	1.0	0.0
66	PLAC	4.0	3.0	2.0	4.0	3.0	2.0	3.0	2.0	2.0	3.0	2.0	1.0	2.0	1.0	1.0	3.0	2.0	1.0
67	DIM	3.0	2.0	1.0	2.0	1.0	0.0	4.0	2.0	1.0	2.0	1.0	1.0	1.0	0.0	0.0	0.0	0.0	0.0
68	CIN	3.0	3.0	2.0	3.0	2.0	2.0	2.0	2.0	2.0	3.0	2.0	2.0	2.0	2.0	1.0	0.0	0.0	0.0
69	CIN	2.0	2.0	1.0	2.0	2.0	1.0	3.0	2.0	0.0	1.0	0.0	0.0	0.0	0.0	0.0	0.0	0.0	0.0
70	PLAC	3.0	3.0	2.0	3.0	3.0	2.0	2.0	2.0	2.0	3.0	3.0	2.0	2.0	2.0	2.0	3.0	3.0	3.0
71	ARL	3.0	1.0	0.0	3.0	2.0	0.0	4.0	2.0	1.0	3.0	2.0	0.0	1.0	0.0	0.0	0.0	0.0	0.0
72	ARL	3.0	2.0	0.0	3.0	2.0	0.0	2.0	1.0	1.0	3.0	2.0	0.0	2.0	1.0	0.0	2.0	1.0	0.0
73	ARL	3.0	2.0	1.0	2.0	1.0	0.0	4.0	2.0	0.0	3.0	2.0	0.0	0.0	0.0	0.0	1.0	0.0	0.0
74	ARL	3.0	2.0	1.0	3.0	2.0	1.0	2.0	1.0	0.0	2.0	1.0	0.0	0.0	0.0	0.0	1.0	0.0	0.0
75	ARL	3.0	2.0	1.0	3.0	1.0	0.0	2.0	1.0	0.0	3.0	2.0	0.0	2.0	1.0	1.0	4.0	2.0	0.0
76	ARL	3.0	3.0	2.0	3.0	3.0	2.0	2.0	2.0	1.0	3.0	2.0	1.0	2.0	2.0	0.0	1.0	1.0	0.0
77	ARL	3.0	2.0	1.0	3.0	2.0	1.0	2.0	1.0	0.0	3.0	2.0	0.0	1.0	0.0	0.0	3.0	2.0	1.0
78	DIM	3.0	2.0	1.0	2.0	2.0	1.0	4.0	3.0	2.0	3.0	2.0	1.0	2.0	2.0	1.0	0.0	0.0	0.0
79	PLAC	3.0	3.0	2.0	3.0	3.0	2.0	4.0	3.0	2.0	2.0	2.0	1.0	0.0	0.0	0.0	2.0	1.0	1.0
80	PLAC	2.0	2.0	2.0	2.0	2.0	1.0	2.0	1.0	1.0	2.0	1.0	1.0	0.0	0.0	0.0	0.0	0.0	0.0
83	CIN	4.0	3.0	2.0	2.0	1.0	0.0	1.0	1.0	0.0	4.0	4.0	1.0	0.0	0.0	0.0	0.0	0.0	0.0
84	CIN	3.5	2.5	1.0	3.5	2.5	1.0	3.5	2.5	1.0	3.5	2.5	1.0	0.0	0.0	0.0	0.0	0.0	0.0
85	CIN	2.5	1.0	0.0	2.5	1.0	0.0	2.5	1.5	0.0	1.0	1.0	0.0	0.0	0.0	0.0	0.0	0.0	0.0
86	ARL	4.0	4.0	4.0	4.0	4.0	4.0	3.0	3.0	3.0	4.0	4.0	4.0	0.0	0.0	0.0	2.5	2.5	2.5
88	ARL	4.0	2.0	0.0	4.0	2.0	1.0	0.0	0.0	0.0	3.0	1.0	0.0	0.0	0.0	0.0	0.0	0.0	0.0
89	ARL	4.0	4.0	2.0	3.0	3.0	1.0	0.0	0.0	0.0	2.0	2.0	1.0	0.0	0.0	0.0	0.0	0.0	0.0
90	ARL	3.0	3.0	3.0	3.0	3.0	3.0	0.0	0.0	0.0	2.0	2.0	2.0	0.0	0.0	0.0	0.0	0.0	0.0
91	DIM	2.0	2.0	0.0	2.0	2.0	0.0	0.0	0.0	0.0	0.0	0.0	0.0	2.0	2.0	0.0	0.0	0.0	0.0
92	CIN	3.0	3.0	3.0	3.0	3.0	3.0	1.0	1.0	1.0	2.0	2.0	2.0	0.0	0.0	0.0	0.0	0.0	0.0
93	PLAC	4.0	4.0	3.0	4.0	4.0	3.0	3.0	3.0	2.0	3.0	3.0	2.0	0.0	0.0	0.0	0.0	0.0	0.0
94	PLAC	4.0	4.0	3.0	4.0	4.0	3.0	3.0	3.0	3.0	3.0	3.0	2.0	0.0	0.0	0.0	0.0	0.0	0.0
95	ARL	4.0	2.0	1.0	4.0	2.0	1.0	0.0	0.0	0.0	3.0	1.0	1.0	0.0	0.0	0.0	0.0	0.0	0.0
96	CIN	3.0	2.0	1.0	1.0	1.0	1.0	4.0	3.0	2.0	0.0	0.0	0.0	3.0	2.0	1.0	0.0	0.0	0.0
97	CIN	4.0	4.0	3.0	4.0	4.0	3.0	3.0	3.0	3.0	0.0	0.0	0.0	0.0	0.0	0.0	0.0	0.0	0.0
98	DIM	4.0	3.0	2.5	4.0	3.0	2.5	0.0	0.0	0.0	3.0	3.0	1.0	0.0	0.0	0.0	0.0	0.0	0.0
99	DIM	4.0	2.0	0.0	4.0	2.0	0.0	2.0	2.0	0.0	3.0	1.0	0.0	4.0	2.0	0.0	0.0	0.0	0.0
100	CIN	3.0	1.5	1.0	3.0	1.5	1.0	0.0	0.0	0.0	0.0	0.0	0.0	0.0	0.0	0.0	0.0	0.0	0.0
101	CIN	4.0	3.0	2.0	4.0	3.0	2.0	3.0	2.0	1.0	0.0	0.0	0.0	2.0	2.0	1.0	3.0	2.0	1.0
102	CIN	2.0	1.0	0.0	4.0	3.0	2.0	3.0	2.0	1.0	3.0	2.0	1.0	0.0	0.0	0.0	0.0	0.0	0.0
103	ARL	3.0	1.0	0.0	4.0	2.0	0.0	2.0	1.0	0.0	0.0	0.0	0.0	0.0	0.0	0.0	0.0	0.0	0.0
104	DIM	2.0	1.0	1.0	0.0	0.0	0.0	0.0	0.0	0.0	0.0	0.0	0.0	0.0	0.0	0.0	0.0	0.0	0.0
105	ARL	4.0	3.0	2.0	4.0	3.0	2.0	3.0	2.0	2.0	3.0	2.0	2.0	3.0	2.0	2.0	0.0	0.0	0.0
106	ARL	3.0	2.0	0.0	3.0	1.0	0.0	0.0	0.0	0.0	3.0	1.0	0.0	3.0	1.0	0.0	0.0	0.0	0.0
107	ARL	4.0	2.0	0.0	3.0	2.0	0.0	0.0	0.0	0.0	2.0	0.0	0.0	3.0	0.0	0.0	1.0	0.0	0.0
108	DIM	4.0	2.0	1.0	4.0	2.0	1.0	1.0	0.0	0.0	3.0	1.0	1.0	2.0	2.0	1.0	0.0	0.0	0.0
109	DIM	4.0	3.0	2.0	4.0	3.0	2.0	2.0	2.0	2.0	4.0	3.0	2.0	2.0	2.0	1.0	0.0	0.0	0.0
110	CIN	4.0	4.0	3.0	4.0	4.0	3.0	3.0	3.0	2.0	3.0	3.0	2.0	2.0	2.0	1.0	0.0	0.0	0.0
111	PLAC	3.0	2.0	2.0	3.0	3.0	3.0	3.0	3.0	2.0	2.0	1.0	1.0	0.0	0.0	0.0	0.0	0.0	0.0
112	PLAC	3.0	3.0	2.0	3.0	2.0	1.0	2.0	2.0	2.0	2.0	1.0	1.0	3.0	3.0	3.0	0.0	0.0	0.0
113	CIN	4.0	3.0	2.0	4.0	3.0	3.0	3.0	3.0	2.0	4.0	3.0	3.0	0.0	0.0	0.0	0.0	0.0	0.0
114	CIN	4.0	2.0	1.0	4.0	2.0	1.0	3.0	2.0	2.0	0.0	0.0	0.0	0.0	0.0	0.0	0.0	0.0	0.0
115	ARL	4.0	2.0	0.0	4.0	2.0	0.0	3.0	1.0	0.0	3.0	1.0	0.0	0.0	0.0	0.0	0.0	0.0	0.0
116	DIM	3.0	2.0	1.0	3.0	2.0	1.0	3.0	2.0	1.0	3.0	2.0	1.0	0.0	0.0	0.0	0.0	0.0	0.0
117	CIN	4.0	4.0	2.0	4.0	4.0	2.0	0.0	0.0	0.0	3.0	3.0	1.0	0.0	0.0	0.0	4.0	4.0	3.0
118	DIM	3.0	2.0	2.0	3.0	2.0	2.0	0.0	0.0	0.0	2.0	1.0	1.0	2.0	1.0	1.0	0.0	0.0	0.0
119	CIN	2.0	2.0	2.0	3.0	3.0	3.0	3.0	3.0	3.0	3.0	3.0	3.0	0.0	0.0	0.0	3.0	3.0	3.0
120	PLAC	4.0	3.0	2.0	2.0	1.0	0.0	2.0	2.0	1.0	2.0	2.0	1.0	0.0	0.0	0.0	0.0	0.0	0.0
121	ARL	4.0	4.0	3.0	4.0	4.0	2.0	0.0	0.0	0.0	2.0	2.0	1.0	0.0	0.0	0.0	0.0	0.0	0.0
122	ARL	4.0	2.0	0.0	4.0	2.0	0.0	4.0	3.0	0.0	2.0	1.0	0.0	0.0	0.0	0.0	0.0	0.0	0.0
123	ARL	4.0	3.0	2.0	4.0	3.0	2.0	0.0	0.0	0.0	3.0	2.0	2.0	0.0	0.0	0.0	0.0	0.0	0.0
124	ARL	4.0	3.0	1.0	3.0	2.0	1.0	3.0	2.0	1.0	3.0	2.0	1.0	0.0	0.0	0.0	0.0	0.0	0.0
125	ARL	3.0	2.0	2.0	3.0	2.0	2.0	3.0	2.0	2.0	2.0	2.0	2.0	0.0	0.0	0.0	0.0	0.0	0.0
126	DIM	3.0	2.0	2.0	3.0	2.0	2.0	3.0	2.0	2.0	3.0	2.0	2.0	3.0	2.0	2.0	3.0	2.0	2.0
127	CIN	3.0	2.0	1.0	3.0	2.0	1.0	3.0	2.0	1.0	2.0	1.0	1.0	0.0	0.0	0.0	0.0	0.0	0.0
128	PLAC	3.0	2.0	2.0	3.0	2.0	1.0	2.0	2.0	1.0	2.0	2.0	2.0	1.0	1.0	1.0	1.0	1.0	1.0
129	PLAC	4.0	4.0	3.0	4.0	4.0	3.0	4.0	4.0	3.0	3.0	3.0	3.0	0.0	0.0	0.0	0.0	0.0	0.0
130	CIN	4.0	2.0	2.0	4.0	2.0	2.0	4.0	2.0	2.0	3.0	2.0	2.0	0.0	0.0	0.0	0.0	0.0	0.0
131	CIN	4.0	4.0	4.0	4.0	4.0	4.0	3.0	3.0	3.0	3.0	3.0	3.0	3.0	3.0	3.0	0.0	0.0	0.0
132	ARL	3.0	2.0	1.0	3.0	3.0	2.0	3.0	2.0	1.0	1.0	1.0	0.0	0.0	0.0	0.0	0.0	0.0	0.0
133	DIM	2.0	2.0	2.0	2.0	2.0	1.0	1.0	1.0	1.0	3.0	2.0	2.0	3.0	3.0	2.0	1.0	1.0	1.0
134	CIN	4.0	3.0	2.0	4.0	3.0	2.0	3.0	2.0	1.0	3.0	1.0	1.0	0.0	0.0	0.0	0.0	0.0	0.0

136	CIN	2.0	2.0	1.0	2.0	2.0	0.0	0.0	0.0	0.0	0.0	1.0	0.0	2.0	0.0	0.0	0.0	0.0	0.0
137	PLAC	4.0	4.0	4.0	4.0	4.0	4.0	3.0	3.0	3.0	4.0	4.0	4.0	3.0	3.0	3.0	3.0	3.0	3.0
138	PLAC	3.0	3.0	0.0	3.0	3.0	0.0	3.0	3.0	0.0	0.0	0.0	0.0	0.0	0.0	0.0	3.0	3.0	0.0
139	PLAC	4.0	4.0	4.0	4.0	4.0	4.0	0.0	0.0	0.0	4.0	4.0	4.0	0.0	0.0	0.0	0.0	0.0	0.0
140	PLAC	4.0	4.0	4.0	4.0	4.0	4.0	3.0	3.0	3.0	3.0	3.0	3.0	2.0	2.0	2.0	2.0	2.0	2.0
141	ARL	4.0	4.0	4.0	4.0	4.0	4.0	3.0	4.0	4.0	2.0	2.0	2.0	0.0	0.0	0.0	0.0	0.0	0.0
142	PLAC	4.0	4.0	4.0	4.0	4.0	4.0	3.0	3.0	3.0	1.0	1.0	1.0	0.0	0.0	0.0	0.0	0.0	0.0
143	ARL	3.0	3.0	2.0	3.0	3.0	1.0	3.0	3.0	1.0	0.0	0.0	0.0	0.0	0.0	0.0	0.0	0.0	0.0
145	CIN	2.0	1.0	1.0	2.0	1.0	1.0	0.0	0.0	0.0	1.0	1.0	1.0	3.0	2.0	1.0	1.0	1.0	1.0
146	PLAC	2.0	2.0	2.0	1.0	1.0	1.0	1.0	1.0	1.0	0.0	0.0	0.0	0.0	0.0	0.0	2.0	2.0	2.0
147	DIM	2.0	2.0	2.0	4.0	4.0	4.0	2.0	2.0	2.0	1.0	1.0	1.0	2.0	2.0	2.0	0.0	0.0	0.0
148	DIM	2.0	1.0	0.0	3.0	1.0	0.0	4.0	2.0	0.0	4.0	3.0	0.0	0.0	0.0	0.0	0.0	0.0	0.0
149	PLAC	4.0	4.0	3.0	2.0	2.0	2.0	2.0	2.0	2.0	3.0	2.0	2.0	3.0	2.0	2.0	0.0	0.0	0.0
150	ARL	4.0	2.0	0.0	4.0	2.0	0.0	0.0	0.0	0.0	0.0	0.0	0.0	1.0	0.0	0.0	2.0	1.0	0.0
151	DIM	3.0	3.0	2.0	4.0	4.0	2.0	4.0	4.0	2.0	4.0	4.0	2.0	0.0	0.0	0.0	0.0	0.0	0.0
152	ARL	4.0	2.0	0.0	4.0	2.0	0.0	4.0	2.0	0.0	3.0	2.0	0.0	0.0	0.0	0.0	0.0	0.0	0.0
153	DIM	3.0	2.0	2.0	3.0	2.0	1.0	4.0	2.0	1.0	4.0	3.0	1.0	3.0	2.0	1.0	0.0	0.0	0.0
154	PLAC	4.0	3.0	3.0	4.0	3.0	3.0	3.0	3.0	3.0	3.0	3.0	3.0	4.0	3.0	3.0	0.0	0.0	0.0
155	PLAC	2.0	1.0	1.0	2.0	1.0	1.0	3.0	1.0	1.0	0.0	0.0	0.0	0.0	0.0	0.0	0.0	0.0	0.0
156	PLAC	2.0	2.0	2.0	3.0	3.0	3.0	2.0	2.0	2.0	0.0	0.0	0.0	1.0	1.0	1.0	0.0	0.0	0.0
157	PLAC	2.0	1.0	1.0	3.0	2.0	1.0	0.0	0.0	0.0	2.0	2.0	1.0	0.0	0.0	0.0	0.0	0.0	0.0
158	PLAC	1.0	1.0	1.0	3.0	3.0	3.0	2.0	2.0	2.0	0.0	0.0	0.0	3.0	3.0	3.0	0.0	0.0	0.0
159	PLAC	2.0	2.0	1.0	3.0	2.0	1.0	3.0	2.0	1.0	3.0	2.0	1.0	2.0	2.0	1.0	2.0	2.0	1.0
160	DIM	2.0	2.0	1.0	1.0	1.0	1.0	3.0	3.0	2.0	1.0	1.0	1.0	4.0	4.0	3.0	0.0	0.0	0.0
161-P	DIM	4.0	3.0	3.0	3.0	2.0	2.0	3.0	2.0	2.0	2.0	2.0	2.0	0.0	0.0	0.0	0.0	0.0	0.0
162-P	DIM	3.0	3.0	2.0	3.0	3.0	2.0	3.0	3.0	2.0	2.0	2.0	2.0	2.0	2.0	1.0	0.0	0.0	0.0
163-P	DIM	3.0	3.0	3.0	3.0	3.0	3.0	2.0	2.0	2.0	0.0	0.0	0.0	0.0	0.0	0.0	0.0	0.0	0.0
164-P	CIN	4.0	3.0	2.0	4.0	3.0	1.0	0.0	0.0	0.0	3.0	3.0	2.0	0.0	0.0	0.0	3.0	3.0	1.0
165-P	DIM	3.0	3.0	2.0	4.0	4.0	3.0	3.0	3.0	2.0	3.0	3.0	1.0	3.0	3.0	2.0	0.0	0.0	0.0
166-P	ARL	3.0	3.0	2.0	3.0	3.0	1.0	4.0	4.0	2.0	0.0	0.0	0.0	3.0	3.0	2.0	0.0	0.0	0.0
167-P	DIM	3.0	3.0	3.0	3.0	3.0	3.0	0.0	0.0	0.0	2.0	2.0	2.0	3.0	3.0	3.0	0.0	0.0	0.0
161-D	DIM	0.0	0.0	0.0	0.0	0.0	0.0	3.0	3.0	3.0	0.0	0.0	0.0	0.0	0.0	0.0	2.0	0.0	0.0
162-D	CIN	2.0	2.0	0.7	1.0	1.7	0.8	1.0	1.0	0.0	1.0	0.8	0.0	3.0	3.0	0.0	0.0	0.0	0.0
163-D	CIN	1.0	1.0	1.0	0.0	0.0	0.0	0.0	0.0	0.0	2.5	2.0	2.0	2.5	2.5	2.5	0.0	0.0	0.0
164-D	CIN	1.0	1.0	0.0	1.0	0.0	0.0	2.0	1.0	0.0	3.0	0.0	0.0	0.0	0.0	0.0	2.0	0.0	0.0
165-D	CIN	0.0	0.0	0.0	0.0	0.0	0.0	4.0	0.0	0.0	0.0	0.0	0.0	2.0	0.0	0.0	0.0	0.0	0.0
166-D	ARL	3.0	3.0	3.0	2.0	0.0	0.0	0.0	0.0	0.0	2.3	1.0	1.0	0.0	0.0	0.0	0.0	0.0	0.0
167-D	DIM	2.0	2.0	2.0	1.5	1.5	1.5	0.8	0.5	0.5	0.6	0.5	0.5	0.0	0.0	0.0	1.0	1.0	1.0
168	CIN	0.0	0.0	0.0	2.0	1.0	0.0	0.0	0.0	0.0	2.0	1.0	0.0	3.0	0.0	0.0	0.0	0.0	0.0
169	DIM	2.0	2.0	1.0	3.0	1.0	1.0	1.0	0.0	1.0	1.0	1.0	0.0	3.0	0.0	1.0	2.0	0.0	2.0
170	CIN	2.5	2.3	2.3	2.0	2.0	2.0	2.0	2.0	2.0	2.0	2.0	2.0	0.0	0.0	0.0	1.0	1.0	1.0
172	ARL	3.0	3.0	1.0	3.0	3.0	1.0	2.0	2.0	1.0	1.0	0.0	0.0	0.0	0.0	0.0	0.0	0.0	0.0
173	ARL	3.0	1.5	0.0	3.0	1.0	1.0	0.0	0.0	0.0	3.0	2.0	2.0	0.0	0.0	0.0	1.0	1.0	0.0
174	ARL	0.0	0.0	0.0	1.0	0.0	0.0	4.0	4.0	2.0	4.0	1.0	0.0	0.0	0.0	0.0	0.0	0.0	0.0
175	ARL	3.0	2.0	0.0	0.0	0.0	0.0	3.0	1.0	0.0	2.0	0.0	0.0	3.0	0.0	0.0	0.0	0.0	0.0
176	PLAC	2.0	2.0	1.0	2.5	2.0	1.0	3.0	2.0	0.0	2.0	2.0	0.0	2.0	2.0	0.0	3.0	3.0	0.0
177	ARL	2.0	1.0	0.0	0.0	0.0	0.0	2.0	1.0	0.0	0.0	0.0	0.0	0.0	0.0	0.0	0.0	0.0	0.0
178	ARL	2.0	1.0	0.5	4.0	0.0	0.0	4.0	1.0	0.0	4.0	0.0	0.0	0.0	0.0	0.0	0.0	0.0	0.0
179	CIN	2.0	0.0	0.0	1.0	0.0	0.0	0.0	1.0	0.0	2.0	1.0	0.0	0.0	0.0	0.0	0.0	0.0	0.0
180	CIN	0.0	0.0	0.0	4.0	3.0	2.0	0.0	0.0	0.0	4.0	2.0	1.0	0.0	0.0	0.0	4.0	3.0	2.0
181	CIN	4.0	4.0	1.0	2.0	2.0	0.0	0.0	0.0	0.0	4.0	3.0	0.0	4.0	0.0	0.0	0.0	0.0	0.0
182	CIN	3.0	2.0	1.0	3.0	2.0	1.0	2.0	0.0	0.0	2.0	0.0	0.0	0.0	0.0	0.0	1.0	1.0	1.0
183	ARL	2.0	0.0	0.0	2.5	0.0	0.0	0.0	0.0	0.0	0.0	0.0	0.0	0.0	0.0	0.0	0.0	0.0	0.0
184	DIM	2.5	2.5	0.0	0.0	0.0	0.0	2.0	0.0	0.0	0.0	0.0	0.0	0.0	0.0	0.0	0.0	0.0	0.0
185	CIN	3.0	3.0	2.0	3.0	3.0	0.0	2.0	2.0	1.0	2.0	2.0	0.0	2.0	2.0	0.0	3.0	0.0	0.0
186	DIM	2.5	2.0	2.0	3.5	3.5	3.0	4.0	4.0	4.0	3.0	3.0	3.0	0.0	0.0	0.0	2.0	2.0	2.0
187	CIN	2.0	1.0	1.0	2.0	2.0	2.0	0.0	0.0	0.0	0.0	0.0	0.0	3.0	3.0	3.0	1.0	1.0	1.0
188	CIN	0.0	0.0	0.0	2.0	2.0	1.0	3.0	2.0	1.0	2.0	3.0	1.0	2.0	2.0	1.0	2.0	1.0	0.0
189	PLAC	3.0	3.0	2.0	2.0	3.0	2.0	3.0	3.0	2.0	3.0	2.0	2.0	2.0	2.0	2.0	3.0	3.0	2.0
190	PLAC	2.0	0.0	0.0	0.0	0.0	0.0	2.0	0.0	0.0	0.0	0.0	0.0	0.0	0.0	0.0	0.0	0.0	0.0
191	PLAC	2.0	2.0	0.8	2.0	2.0	0.6	0.0	1.0	0.0	1.0	2.0	0.5	3.0	2.5	2.0	2.0	2.0	0.0
192	ARL	2.0	0.0	2.0	3.0	0.0	0.0	3.0	0.0	0.0	4.0	0.0	0.0	0.0	0.0	0.0	0.0	0.0	0.0
193	PLAC	2.0	2.0	1.0	2.0	1.0	0.0	3.0	3.0	2.0	0.0	0.0	0.0	0.0	0.0	0.0	0.0	0.0	0.0
194	ARL	4.0	0.0	1.0	3.0	0.0	1.0	0.0	0.0	0.0	4.0	0.0	1.0	4.0	0.0	0.0	0.0	0.0	0.0
195	ARL	3.0	2.0	1.0	2.0	2.0	0.0	1.0	1.5	1.0	4.0	2.0	0.0	0.0	0.0	0.0	2.0	2.0	0.0
196	DIM	2.0	2.0	2.0	1.0	0.0	0.0	3.0	2.0	2.0	4.0	0.0	0.0	2.0	3.0	2.0	3.0	2.0	3.0
197	DIM	2.0	0.0	0.0	2.0	2.0	1.0	2.5	0.0	1.0	2.5	2.5	1.0	0.0	0.0	0.0	2.0	0.0	0.0
198	DIM	2.0	2.0	2.0	2.0	2.0	0.0	0.0	0.0	0.0	0.0	0.0	0.0	2.0	2.0	1.0	2.0	1.0	0.0

199	CIN	2.0	2.0	1.0	0.0	0.0	2.0	2.0	1.0	0.0	0.0	0.0	0.0	4.0	2.0	2.0	3.0	2.0	2.0
200	PLAC	2.0	1.5	0.0	2.0	2.0	0.0	0.0	0.0	0.0	4.0	3.0	0.0	0.0	0.0	0.0	0.0	0.0	0.0
201	DIM	2.0	2.0	2.0	2.0	2.0	2.0	0.0	0.0	0.0	0.0	0.0	0.0	1.0	0.0	0.5	2.0	0.0	1.0
202	CIN	2.0	2.0	2.0	1.0	0.0	0.0	3.0	2.0	2.0	0.0	0.0	0.0	0.0	0.0	0.0	2.0	0.0	3.0
203	DIM	0.0	0.0	0.0	2.0	2.0	0.0	0.0	0.0	0.0	2.0	2.0	0.0	0.0	0.0	0.0	2.0	1.0	0.0
204	CIN	2.0	2.0	0.0	3.0	2.0	0.0	3.0	2.0	0.0	2.0	1.0	1.0	0.0	0.0	0.0	1.0	1.0	0.0
205	CIN	3.0	3.0	3.0	2.5	2.5	2.5	2.5	2.5	1.5	2.0	2.0	2.0	2.0	2.0	2.0	4.0	4.0	3.0
207	PLAC	2.0	2.0	1.0	2.0	2.0	1.0	0.0	0.0	0.0	2.0	2.0	2.0	0.0	0.0	0.0	1.0	1.0	1.0
208	PLAC	1.0	0.0	0.0	1.0	0.0	0.0	2.0	0.0	0.0	2.0	0.0	0.0	0.0	0.0	0.0	0.0	0.0	0.0
209	ARL	4.0	2.0	0.0	4.0	2.0	0.5	4.0	1.0	0.0	0.0	0.0	0.0	0.0	0.0	0.0	2.0	0.0	0.0
210	PLAC	3.0	2.0	1.0	0.0	0.0	0.0	4.0	3.5	2.0	0.0	0.0	0.0	0.0	0.0	0.0	2.0	2.0	2.0
211	ARL	2.0	2.0	2.0	3.0	3.0	0.0	0.0	0.0	0.0	2.5	2.5	0.0	0.0	0.0	0.0	0.0	0.0	0.0
212	PLAC	2.5	3.0	3.0	2.5	2.5	2.5	2.0	2.0	2.0	1.0	0.0	0.0	2.0	1.0	1.0	2.0	2.0	2.0
213	DIM	2.0	2.0	2.0	3.0	3.0	3.0	4.0	4.0	3.0	4.0	3.0	0.0	0.0	0.0	0.0	0.0	0.0	0.0
214	DIM	3.0	2.0	0.0	0.0	0.0	0.0	0.0	0.0	0.0	0.0	0.0	0.0	3.0	2.0	0.0	0.0	0.0	0.0
215	DIM	2.0	1.0	0.0	2.0	1.0	0.0	3.0	2.0	0.0	3.0	2.0	0.0	0.0	0.0	0.0	2.0	1.0	0.0
216	DIM	4.0	0.0	0.0	4.0	0.0	0.0	2.5	0.0	0.0	4.0	0.0	0.0	0.0	0.0	0.0	3.0	0.0	0.0
217	PLAC	3.0	2.0	2.0	2.0	1.0	1.0	4.0	2.0	2.0	2.0	0.0	0.0	0.0	0.0	0.0	0.0	0.0	0.0
218	DIM	4.0	4.0	4.0	4.0	4.0	4.0	3.0	3.0	3.0	0.0	0.0	0.0	1.0	1.0	0.0	3.0	3.0	3.0
219	CIN	3.0	3.0	3.0	3.0	3.0	3.0	2.0	2.0	1.0	3.0	3.0	3.0	2.0	2.0	1.0	3.0	3.5	3.0
220	DIM	3.0	3.0	1.0	1.0	1.0	0.0	2.0	2.0	2.0	0.0	0.0	0.0	2.0	2.0	1.0	3.0	3.0	2.0
221	CIN	3.0	3.0	1.0	2.0	1.0	0.0	1.0	0.0	0.0	0.0	0.0	0.0	0.0	0.0	0.0	0.0	0.0	0.0
222	DIM	3.0	3.0	3.0	2.0	2.0	2.0	2.0	2.0	2.0	3.0	3.0	3.0	2.0	2.0	0.0	1.0	0.0	0.0
223	PLAC	2.0	2.0	2.0	2.0	2.0	1.0	3.0	3.0	1.0	2.0	2.0	0.0	2.0	2.0	0.0	2.0	2.0	1.0
224	PLAC	2.5	2.0	1.0	0.0	0.0	0.0	2.5	2.0	1.0	0.0	0.0	0.0	0.0	0.0	0.0	0.0	0.0	0.0
225	PLAC	2.0	1.0	0.0	2.0	1.6	1.0	2.0	0.0	1.0	1.5	1.5	1.0	1.0	0.0	1.0	2.0	1.0	0.0
226	ARL	1.0	0.0	0.0	1.0	0.5	0.5	3.0	0.0	0.0	2.0	0.0	0.0	0.0	0.0	0.0	0.0	0.0	0.0
227	PLAC	1.5	1.5	1.0	1.5	1.5	1.0	3.0	3.0	2.0	0.0	0.0	0.0	0.0	0.0	0.0	0.0	0.0	0.0
228	ARL	2.0	2.0	0.0	2.0	2.0	0.0	2.0	2.0	0.0	2.0	1.5	0.0	1.0	1.0	0.0	3.0	3.0	0.0
229	PLAC	3.0	3.0	1.0	3.0	3.0	1.0	0.0	0.0	0.0	3.0	3.0	1.0	2.0	2.0	1.0	2.0	2.0	0.0
230	DIM	3.0	3.0	2.0	3.0	3.0	2.0	3.0	3.0	2.0	2.0	2.0	2.0	0.0	0.0	0.0	2.0	3.0	2.0
231	ARL	4.0	3.0	0.0	3.0	2.0	0.0	0.0	0.0	0.0	4.0	3.0	0.0	0.0	0.0	0.0	2.0	1.0	0.0
233	DIM	2.5	2.5	2.5	2.5	3.0	3.0	1.0	0.0	0.0	0.0	0.0	0.0	0.0	0.0	0.0	0.0	0.0	0.0
234	PLAC	2.5	2.5	0.5	3.0	2.0	1.0	2.0	2.0	1.0	2.0	2.0	1.0	0.0	0.0	0.0	2.0	2.0	1.0
235	ARL	3.0	2.0	1.0	3.0	2.0	0.0	0.0	0.0	0.0	2.0	2.0	2.0	3.0	2.0	0.0	4.0	2.0	0.0
236	CIN	0.0	0.0	0.0	0.0	0.0	0.0	3.0	3.0	1.0	0.0	0.0	0.0	0.0	0.0	0.0	0.0	0.0	0.0
237	ARL	2.0	1.0	0.5	3.0	2.0	1.0	2.0	2.0	2.0	2.0	1.0	1.0	0.0	0.0	0.0	2.0	1.0	0.0
238	ARL	4.0	2.0	3.0	3.0	2.0	2.0	3.0	1.0	0.0	1.0	0.0	0.0	0.0	0.0	0.0	0.0	0.0	0.0
239	ARL	1.0	1.0	0.0	0.0	0.0	0.0	2.0	2.0	0.0	0.0	0.0	0.0	0.0	0.0	0.0	0.0	0.0	0.0
240	PLAC	4.0	3.5	2.0	3.5	3.0	2.0	2.5	2.5	2.0	3.5	3.0	1.0	2.0	2.0	2.0	4.0	4.0	2.0

PATNO: Patient number; N = 239 (ITT population)
MEDICAT (medication): CIN = Cinnarizine 50mg, DIM = Dimenhydrinate 100mg, ARL = Arlevert (Cinnarizine 20mg + Dimenhydrinate 40mg), PLAC = Placebo
Spontaneous vertigo symptoms: Dystasia and walking unsteadiness (DYSTAS), Staggering (STAGGER), Rotary sensation (ROTARY), Tendency to fall (FALL), Lift sensation (LIFT), Blackout (SCOTO)
0 = baseline, 1 = after 1 week, 2 = after 4 weeks of treatment
