# Supplementary material for: The Mean Vertigo Score (MVS) Outcome Scale and Its Use in Clinical Research for Quantifying Vestibular Disorders
Source: Front Neurol. 2021 May 5;12:601749. doi: 10.3389/fneur.2021.601749 (PMC8131667; doi:10.3389/fneur.2021.601749)
Supplement: Supplementary file 2 [file Table_2.pdf]

Supplementary Table 1b: Intensity of vertigo in consequence of 6 triggering factors in the course of 4 weeks of treatment

PATNO	MEDICAT	CHANGE0	CHANGE1	CHANGE2	BOW0	BOW1	BOW2	GETUP0	GETUP1	GETUP2	DRIV0	DRIV1	DRIV2	HEADMOV0	HEADMOV1	HEADMOV2	EYEMOV0	EYEMOV1	EYEMOV2
1	CIN	3.0	3.0	2.0	2.0	2.0	1.0	2.0	2.0	1.0	1.0	0.0	0.0	4.0	3.0	3.0	1.0	1.0	1.0
2	CIN	3.0	2.0	1.0	2.0	1.0	1.0	2.0	1.0	1.0	0.0	0.0	0.0	3.0	2.0	1.0	1.0	1.0	0.0
3	CIN	1.0	1.0	1.0	2.0	1.0	1.0	2.0	0.0	0.0	0.0	0.0	0.0	3.0	1.0	1.0	2.0	0.0	0.0
4	PLAC	2.0	2.0	1.0	1.2	1.0	1.0	3.7	2.0	0.0	0.0	0.0	1.0	0.0	0.0	0.0	0.0	0.0	0.0
5	DIM	1.0	1.0	1.0	1.0	1.0	1.0	1.5	1.5	1.5	0.0	0.0	0.0	0.0	0.0	0.0	0.0	0.0	0.0
6	DIM	3.0	2.0	1.0	2.0	2.0	0.0	0.0	0.0	0.0	0.0	0.0	0.0	2.0	2.0	1.0	0.0	0.0	0.0
7	DIM	0.0	0.0	0.0	1.0	1.0	1.0	2.0	2.0	1.0	0.0	0.0	0.0	1.0	1.0	1.0	0.0	0.0	0.0
8	PLAC	1.0	0.0	0.0	1.0	0.0	0.0	0.0	0.0	0.0	0.0	0.0	0.0	0.0	0.0	0.0	0.0	0.0	0.0
9	PLAC	0.0	0.0	0.0	4.0	3.0	2.0	1.0	0.0	0.0	0.0	0.0	0.0	0.0	0.0	0.0	0.0	0.0	0.0
10	DIM	0.0	0.0	0.0	1.0	1.0	1.0	2.0	1.8	1.8	1.0	1.0	1.0	1.0	1.0	1.0	1.0	1.0	1.0
11	ARL	3.0	0.0	0.0	3.0	0.0	0.0	3.0	0.0	0.0	0.0	0.0	0.0	0.0	0.0	0.0	0.0	0.0	0.0
12	CIN	0.0	0.0	0.0	0.0	0.0	0.0	4.0	4.0	3.0	0.0	0.0	0.0	4.0	4.0	3.0	0.0	0.0	0.0
13	PLAC	3.0	2.0	1.0	2.0	2.0	2.0	2.0	2.0	2.0	3.0	2.0	1.0	3.0	2.0	2.0	3.0	3.0	2.0
14	PLAC	3.0	1.0	1.0	1.0	1.0	0.0	1.0	0.0	0.0	0.0	0.0	0.0	3.0	1.0	0.0	0.0	0.0	0.0
15	DIM	2.0	2.0	1.0	1.0	1.0	1.0	1.0	1.0	1.0	0.0	0.0	0.0	3.0	2.0	2.0	1.0	1.0	1.0
16	PLAC	3.0	3.0	2.0	2.0	2.0	2.0	2.0	2.0	1.0	0.0	0.0	0.0	4.0	3.0	2.0	2.0	2.0	2.0
17	ARL	4.0	2.0	1.0	3.0	2.0	0.0	0.0	0.0	0.0	0.0	0.0	0.0	4.0	2.0	1.0	3.0	2.0	0.0
18	CIN	3.0	3.0	2.0	3.0	3.0	2.0	2.0	2.0	2.0	0.0	0.0	0.0	3.0	2.0	2.0	1.0	0.0	0.0
19	CIN	4.0	3.0	2.0	1.0	1.0	1.0	1.0	1.0	1.0	0.0	0.0	0.0	3.0	2.0	1.0	3.0	2.0	2.0
20	PLAC	3.0	3.0	2.0	2.0	2.0	1.0	2.0	2.0	1.0	0.0	0.0	0.0	3.0	2.0	1.0	2.0	2.0	0.0
21	CIN	1.0	0.0	0.0	2.0	1.0	1.0	0.0	0.0	0.0	0.0	0.0	0.0	0.0	0.0	0.0	0.0	0.0	0.0
22	CIN	3.0	0.0	0.0	0.0	0.0	0.0	3.0	0.0	0.0	0.0	0.0	0.0	3.0	0.0	0.0	0.0	0.0	0.0
23	PLAC	4.0	3.0	2.0	3.0	2.0	1.0	3.0	2.0	0.0	0.0	0.0	0.0	3.0	1.0	1.0	0.0	0.0	0.0
24	CIN	2.0	0.0	0.0	2.0	1.0	0.0	2.0	1.0	0.0	0.0	0.0	0.0	2.0	1.0	0.0	1.0	0.0	0.0
25	PLAC	3.0	2.0	1.0	1.0	1.0	1.0	1.0	1.0	0.0	0.0	0.0	0.0	4.0	2.0	1.0	1.0	0.0	0.0
26	PLAC	3.0	2.0	1.0	2.0	2.0	1.0	2.0	1.0	1.0	0.0	0.0	0.0	3.0	2.0	1.0	2.0	2.0	1.0
27	DIM	2.0	2.0	1.0	1.0	1.0	1.0	1.0	1.0	0.0	0.0	0.0	0.0	3.0	2.0	1.0	2.0	2.0	1.0
28	DIM	3.0	2.0	1.0	3.0	2.0	1.0	2.0	1.0	1.0	1.0	1.0	1.0	3.0	2.0	1.0	2.0	1.0	0.0
29	PLAC	3.0	3.0	2.0	2.0	2.0	2.0	2.0	1.0	1.0	1.0	1.0	1.0	3.0	2.0	1.0	2.0	1.0	1.0
30	CIN	2.0	2.0	2.0	1.0	1.0	0.0	1.0	1.0	1.0	0.0	0.0	0.0	2.0	1.0	1.0	1.0	1.0	0.0
31	ARL	3.0	2.0	0.0	3.0	2.0	0.0	3.0	1.0	0.0	0.0	0.0	0.0	3.0	2.0	0.0	2.0	1.0	0.0
32	DIM	1.0	1.0	0.0	0.0	0.0	0.0	0.0	0.0	0.0	0.0	0.0	0.0	2.0	2.0	1.0	0.0	0.0	0.0
33	PLAC	0.0	0.0	0.0	1.0	0.0	0.0	1.0	0.0	0.0	0.0	0.0	0.0	0.0	0.0	0.0	0.0	0.0	0.0
34	DIM	3.0	2.0	1.0	1.0	1.0	0.0	1.0	1.0	1.0	1.0	0.0	0.0	3.0	2.0	1.0	1.0	0.0	0.0
35	CIN	2.0	2.0	1.0	1.0	1.0	1.0	1.0	0.0	0.0	0.0	0.0	0.0	2.0	1.0	0.0	2.0	1.0	0.0
36	ARL	3.0	2.0	0.0	2.0	1.0	0.0	2.0	2.0	0.0	0.0	0.0	0.0	3.0	2.0	0.0	0.0	0.0	0.0
37	DIM	0.0	0.0	0.0	0.0	0.0	0.0	0.0	0.0	0.0	3.0	3.0	3.0	0.0	0.0	0.0	1.0	1.0	1.0
38	CIN	2.0	2.0	1.0	1.0	1.0	1.0	2.0	2.0	1.0	0.0	0.0	0.0	3.0	2.0	2.0	0.0	0.0	0.0
39	ARL	2.0	1.0	1.0	2.0	1.0	0.0	2.0	1.0	0.0	0.0	0.0	0.0	0.0	0.0	0.0	0.0	0.0	0.0
40	DIM	2.0	3.0	1.0	2.0	3.0	2.0	2.0	3.0	2.0	0.0	0.0	0.0	0.0	0.0	0.0	0.0	0.0	0.0
41	DIM	1.0	1.0	1.0	1.0	1.0	0.0	2.0	1.0	0.0	0.0	0.0	0.0	2.0	2.0	1.0	1.0	1.0	1.0
42	CIN	3.0	2.0	1.0	2.0	2.0	1.0	2.0	2.0	1.0	0.0	0.0	0.0	2.0	1.0	1.0	1.0	1.0	0.0
43	CIN	3.0	2.0	2.0	3.0	2.0	1.0	2.0	1.0	1.0	0.0	0.0	0.0	2.0	2.0	0.0	2.0	1.0	1.0
44	DIM	3.0	3.0	2.0	3.0	2.0	2.0	2.0	2.0	1.0	0.0	0.0	0.0	2.0	1.0	1.0	2.0	2.0	1.0
45	ARL	2.0	2.0	1.0	2.0	1.0	0.0	1.0	1.0	0.0	1.0	1.0	1.0	0.0	0.0	0.0	0.0	0.0	0.0
46	CIN	2.0	1.0	1.0	1.0	1.0	0.0	2.0	2.0	1.0	0.0	0.0	0.0	2.0	1.0	1.0	0.0	0.0	0.0
47	DIM	3.0	2.0	2.0	2.0	1.0	1.0	2.0	1.0	1.0	0.0	0.0	0.0	0.0	0.0	0.0	0.0	0.0	0.0
48	ARL	2.0	1.0	0.0	3.0	2.0	1.0	3.0	2.0	1.0	0.0	0.0	0.0	2.0	1.0	0.0	2.0	1.0	1.0
49	CIN	3.0	2.0	2.0	3.0	2.0	1.0	2.0	1.0	1.0	0.0	0.0	0.0	2.0	1.0	1.0	2.0	1.0	0.0
50	ARL	3.0	2.0	1.0	3.0	3.0	1.0	2.0	2.0	2.0	0.0	0.0	0.0	4.0	3.0	1.0	3.0	2.0	1.0
51	DIM	3.0	3.0	2.0	3.0	3.0	2.0	3.0	2.0	1.0	0.0	0.0	0.0	2.0	1.0	0.0	2.0	2.0	0.0
52	ARL	3.0	2.0	1.0	3.0	2.0	1.0	2.0	2.0	1.0	0.0	0.0	0.0	3.0	2.0	1.0	1.0	0.0	0.0
53	ARL	3.0	1.0	0.0	3.0	2.0	0.0	2.0	0.0	0.0	0.0	0.0	0.0	3.0	0.0	0.0	2.0	0.0	0.0
54	ARL	1.0	1.0	1.0	1.0	1.0	0.0	1.0	1.0	0.0	0.0	0.0	0.0	2.0	1.0	1.0	1.0	1.0	0.0
55	CIN	1.0	0.0	0.0	0.0	0.0	0.0	2.0	0.0	0.0	2.0	1.0	0.0	2.0	0.0	0.0	1.0	0.0	0.0
56	DIM	2.0	2.0	0.0	2.0	1.0	1.0	1.0	0.0	0.0	0.0	0.0	0.0	3.0	2.0	2.0	1.0	1.0	0.0
57	DIM	3.0	3.0	2.0	3.0	3.0	1.0	2.0	1.0	1.0	0.0	0.0	0.0	2.0	1.0	0.0	1.0	0.0	0.0
58	PLAC	3.0	3.0	1.0	2.0	2.0	1.0	2.0	2.0	1.0	0.0	0.0	0.0	3.0	2.0	1.0	2.0	2.0	1.0
59	PLAC	3.0	2.0	1.0	3.0	2.0	1.0	2.0	2.0	1.0	0.0	0.0	0.0	2.0	1.0	0.0	2.0	1.0	0.0
60	ARL	3.0	3.0	2.0	2.0	2.0	1.0	2.0	1.0	1.0	0.0	0.0	0.0	3.0	2.0	1.0	2.0	2.0	1.0
61	PLAC	3.0	3.0	1.0	1.0	1.0	0.0	1.0	1.0	0.0	0.0	0.0	0.0	2.0	1.0	0.0	1.0	0.0	0.0
62	ARL	3.0	1.0	0.0	3.0	1.0	0.0	2.0	1.0	0.0	0.0	0.0	0.0	2.0	0.0	0.0	2.0	0.0	0.0
63	PLAC	3.0	3.0	2.0	3.0	2.0	2.0	3.0	2.0	2.0	0.0	0.0	0.0	2.0	2.0	2.0	2.0	1.0	1.0
64	DIM	1.0	1.0	0.0	1.0	1.0	1.0	1.0	1.0	0.0	0.0	0.0	0.0	2.0	1.0	0.0	1.0	1.0	0.0
65	CIN	3.0	3.0	2.0	3.0	2.0	1.0	2.0	2.0	1.0	0.0	0.0	0.0	2.0	2.0	1.0	2.0	1.0	0.0
66	PLAC	3.0	2.0	2.0	3.0	2.0	2.0	2.0	1.0	1.0	0.0	0.0	0.0	2.0	1.0	1.0	2.0	1.0	1.0
67	DIM	2.0	1.0	1.0	2.0	1.0	0.0	1.0	0.0	0.0	0.0	0.0	0.0	1.0	0.0	0.0	2.0	1.0	1.0
68	CIN	2.0	2.0	1.0	1.0	0.0	0.0	1.0	1.0	0.0	0.0	0.0	0.0	2.0	2.0	1.0	2.0	2.0	2.0
69	CIN	3.0	2.0	1.0	2.0	2.0	1.0	2.0	2.0	1.0	0.0	0.0	0.0	3.0	2.0	0.0	0.0	0.0	0.0

70	PLAC	3.0	2.0	2.0	2.0	2.0	1.0	2.0	2.0	2.0	0.0	0.0	0.0	2.0	2.0	1.0	2.0	1.0	1.0
71	ARL	3.0	1.0	0.0	2.0	0.0	0.0	2.0	1.0	0.0	0.0	0.0	0.0	2.0	1.0	0.0	0.0	0.0	0.0
72	ARL	3.0	2.0	0.0	2.0	2.0	0.0	2.0	1.0	0.0	0.0	0.0	0.0	3.0	2.0	0.0	2.0	1.0	0.0
73	ARL	3.0	2.0	0.0	2.0	1.0	0.0	3.0	2.0	1.0	0.0	0.0	0.0	3.0	2.0	0.0	2.0	1.0	0.0
74	ARL	2.0	1.0	1.0	2.0	1.0	1.0	1.0	1.0	1.0	0.0	0.0	0.0	2.0	1.0	1.0	1.0	1.0	0.0
75	ARL	3.0	2.0	0.0	3.0	1.0	0.0	2.0	2.0	1.0	0.0	0.0	0.0	3.0	2.0	0.0	2.0	1.0	0.0
76	ARL	3.0	2.0	1.0	3.0	3.0	2.0	2.0	2.0	1.0	0.0	0.0	0.0	3.0	2.0	1.0	2.0	1.0	0.0
77	ARL	3.0	2.0	0.0	2.0	1.0	0.0	2.0	1.0	1.0	0.0	0.0	0.0	2.0	1.0	0.0	2.0	1.0	0.0
78	DIM	2.0	2.0	1.0	1.0	1.0	1.0	1.0	0.0	0.0	0.0	0.0	0.0	0.0	0.0	0.0	1.0	0.0	0.0
79	PLAC	2.0	1.0	1.0	1.0	0.0	0.0	1.0	0.0	0.0	0.0	0.0	0.0	3.0	1.0	1.0	2.0	1.0	1.0
80	PLAC	2.0	2.0	1.0	1.0	1.0	1.0	1.0	1.0	0.0	0.0	0.0	0.0	0.0	0.0	0.0	0.0	0.0	0.0
83	CIN	0.0	0.0	0.0	4.0	4.0	3.0	4.0	4.0	3.0	0.0	0.0	0.0	0.0	0.0	0.0	0.0	0.0	0.0
84	CIN	0.0	0.0	0.0	0.0	0.0	0.0	0.0	0.0	0.0	0.0	0.0	0.0	0.0	0.0	0.0	0.0	0.0	0.0
85	CIN	4.0	2.0	0.0	4.0	2.0	0.0	0.0	0.0	0.0	0.0	0.0	0.0	4.0	2.0	0.0	0.0	0.0	0.0
86	ARL	4.0	4.0	4.0	4.0	4.0	4.0	4.0	4.0	4.0	0.0	0.0	0.0	4.0	4.0	4.0	0.0	0.0	0.0
88	ARL	3.0	1.8	1.0	3.0	2.0	0.0	3.0	2.0	0.0	0.0	0.0	0.0	3.0	1.0	0.0	0.0	0.0	0.0
89	ARL	4.0	4.0	2.0	4.0	4.0	3.0	4.0	4.0	2.0	0.0	0.0	0.0	4.0	4.0	2.0	0.0	0.0	0.0
90	ARL	2.0	2.0	2.0	0.0	0.0	0.0	0.0	0.0	0.0	0.0	0.0	0.0	2.0	2.0	2.0	0.0	0.0	0.0
91	DIM	4.0	2.0	1.0	4.0	2.0	1.0	4.0	2.0	1.0	0.0	0.0	0.0	4.0	2.0	0.0	3.0	1.0	0.0
92	CIN	4.0	4.0	4.0	4.0	4.0	4.0	4.0	4.0	4.0	0.0	0.0	0.0	4.0	4.0	4.0	0.0	0.0	0.0
93	PLAC	0.0	0.0	0.0	0.0	0.0	0.0	0.0	0.0	0.0	3.0	2.0	2.0	3.0	2.0	2.0	0.0	0.0	0.0
94	PLAC	3.0	3.0	2.0	2.0	2.0	2.0	2.0	2.0	1.0	0.0	0.0	0.0	2.0	2.0	1.0	3.0	3.0	2.0
95	ARL	4.0	2.0	1.0	0.0	0.0	0.0	4.0	2.0	1.0	0.0	0.0	0.0	3.0	2.0	1.0	0.0	0.0	0.0
96	CIN	4.0	3.0	2.0	0.0	0.0	0.0	0.0	0.0	0.0	0.0	0.0	0.0	3.0	2.0	1.0	0.0	0.0	0.0
97	CIN	3.0	3.0	3.0	2.0	2.0	2.0	2.0	2.0	2.0	0.0	0.0	0.0	0.0	0.0	0.0	0.0	0.0	0.0
98	DIM	4.0	2.0	2.0	2.0	2.0	1.0	0.0	1.0	1.0	0.0	0.0	0.0	0.0	0.0	0.0	0.0	0.0	0.0
99	DIM	4.0	2.0	0.0	4.0	2.0	0.0	4.0	2.0	0.0	0.0	0.0	0.0	3.0	1.0	0.0	0.0	0.0	0.0
100	CIN	3.0	2.0	2.0	3.0	1.0	1.0	3.0	1.5	1.0	0.0	0.0	0.0	4.0	2.5	2.0	0.0	0.0	0.0
101	CIN	4.0	3.0	2.0	4.0	3.0	1.0	3.0	2.0	1.0	0.0	0.0	0.0	3.0	2.0	1.0	0.0	0.0	0.0
102	CIN	4.0	3.0	2.0	0.0	0.0	0.0	3.0	2.0	1.0	0.0	0.0	0.0	4.0	3.0	2.0	0.0	0.0	0.0
103	ARL	3.0	2.0	0.0	0.0	0.0	0.0	3.0	2.0	0.0	0.0	0.0	0.0	0.0	0.0	0.0	0.0	0.0	0.0
104	DIM	4.0	2.0	2.0	4.0	2.0	2.0	4.0	2.0	2.0	0.0	0.0	0.0	4.0	2.0	0.0	0.0	0.0	0.0
105	ARL	3.0	2.0	1.0	3.0	2.0	1.0	3.0	2.0	1.0	0.0	0.0	0.0	3.0	2.0	0.0	2.0	1.0	1.0
106	ARL	0.0	0.0	0.0	3.0	1.0	0.0	3.0	1.0	0.0	0.0	0.0	0.0	3.0	1.0	0.0	0.0	0.0	0.0
107	ARL	3.0	1.0	0.0	3.0	1.0	0.0	3.0	1.0	0.0	3.0	1.0	0.0	3.0	1.0	0.0	3.0	0.0	0.0
108	DIM	3.0	1.0	1.0	3.0	1.0	1.0	3.0	2.0	1.0	0.0	0.0	0.0	3.0	1.0	0.0	0.0	0.0	0.0
109	DIM	4.0	3.0	2.0	4.0	3.0	2.0	4.0	3.0	2.0	0.0	0.0	0.0	3.0	2.0	2.0	0.0	0.0	0.0
110	CIN	4.0	4.0	2.0	4.0	4.0	3.0	3.0	3.0	2.0	3.0	3.0	2.0	2.0	2.0	2.0	0.0	0.0	0.0
111	PLAC	3.0	3.0	2.0	3.0	3.0	2.0	3.0	3.0	2.0	3.0	3.0	2.0	0.0	0.0	0.0	0.0	0.0	0.0
112	PLAC	4.0	3.0	2.0	4.0	3.0	2.0	3.0	3.0	2.0	2.0	2.0	1.0	3.0	3.0	2.0	3.0	2.0	1.0
113	CIN	4.0	3.0	3.0	4.0	3.0	3.0	3.0	3.0	3.0	0.0	0.0	0.0	3.0	3.0	3.0	0.0	0.0	0.0
114	CIN	4.0	3.0	1.0	4.0	3.0	1.0	3.0	2.0	1.0	0.0	0.0	0.0	0.0	0.0	0.0	0.0	0.0	0.0
115	ARL	3.0	1.0	0.0	3.0	1.0	0.0	3.0	2.0	0.0	0.0	0.0	0.0	2.0	1.0	0.0	0.0	0.0	0.0
116	DIM	3.0	2.0	0.0	2.0	2.0	1.0	2.0	1.0	1.0	0.0	0.0	0.0	2.0	1.0	1.0	0.0	0.0	0.0
117	CIN	3.0	2.0	1.0	3.0	2.0	1.0	2.0	2.0	1.0	0.0	0.0	0.0	0.0	0.0	0.0	0.0	0.0	0.0
118	DIM	0.0	0.0	0.0	2.0	2.0	1.0	2.0	2.0	1.0	0.0	0.0	0.0	2.0	2.0	1.0	0.0	0.0	0.0
119	CIN	2.0	2.0	2.0	2.0	2.0	2.0	2.0	2.0	2.0	3.0	3.0	3.0	0.0	0.0	0.0	2.0	2.0	2.0
120	PLAC	3.0	2.0	1.0	3.0	2.0	1.0	3.0	2.0	1.0	0.0	0.0	0.0	3.0	1.0	0.0	0.0	0.0	0.0
121	ARL	3.0	3.0	2.0	3.0	3.0	2.0	0.0	0.0	0.0	0.0	0.0	0.0	2.0	2.0	2.0	0.0	0.0	0.0
122	ARL	3.0	2.0	0.0	3.0	2.0	0.0	3.0	2.0	0.0	0.0	0.0	0.0	2.0	1.0	0.0	0.0	0.0	0.0
123	ARL	4.0	3.0	2.0	0.0	0.0	0.0	0.0	0.0	0.0	2.0	1.0	1.0	3.0	2.0	2.0	0.0	0.0	0.0
124	ARL	4.0	3.0	1.0	4.0	3.0	1.0	4.0	3.0	1.0	0.0	0.0	0.0	3.0	2.0	2.0	0.0	0.0	0.0
125	ARL	0.0	0.0	0.0	0.0	0.0	0.0	0.0	0.0	0.0	0.0	0.0	0.0	0.0	0.0	0.0	0.0	0.0	0.0
126	DIM	4.0	2.0	1.0	4.0	2.0	1.0	4.0	2.0	1.0	0.0	0.0	0.0	3.0	2.0	2.0	0.0	0.0	0.0
127	CIN	3.0	2.0	2.0	3.0	2.0	2.0	2.0	1.0	1.0	0.0	0.0	0.0	3.0	1.0	1.0	2.0	1.0	1.0
128	PLAC	3.0	2.0	1.0	3.0	2.0	1.0	2.0	2.0	1.0	2.0	2.0	1.0	2.0	2.0	1.0	2.0	2.0	1.0
129	PLAC	2.0	2.0	1.0	2.0	2.0	2.0	0.0	0.0	0.0	0.0	0.0	0.0	0.0	0.0	0.0	0.0	0.0	0.0
130	CIN	0.0	0.0	0.0	0.0	0.0	0.0	0.0	0.0	0.0	0.0	0.0	0.0	2.0	1.0	1.0	0.0	0.0	0.0
131	CIN	4.0	4.0	4.0	4.0	4.0	4.0	4.0	4.0	4.0	0.0	0.0	0.0	3.0	3.0	3.0	0.0	0.0	0.0
132	ARL	4.0	2.0	1.0	4.0	2.0	2.0	4.0	2.0	1.0	0.0	0.0	0.0	3.0	2.0	2.0	0.0	0.0	0.0
133	DIM	2.0	2.0	2.0	3.0	3.0	2.0	3.0	3.0	2.0	0.0	0.0	0.0	0.0	0.0	0.0	1.0	1.0	0.0
134	CIN	4.0	3.0	2.0	4.0	3.0	2.0	4.0	3.0	2.0	2.0	2.0	1.0	3.0	3.0	2.0	0.0	0.0	0.0
136	CIN	3.0	1.0	0.0	3.0	1.0	0.0	3.0	1.0	0.0	1.0	1.0	0.0	3.0	2.0	1.0	3.0	1.0	0.0
137	PLAC	4.0	4.0	4.0	4.0	4.0	4.0	4.0	4.0	4.0	0.0	0.0	0.0	0.0	0.0	0.0	0.0	0.0	0.0
138	PLAC	4.0	4.0	1.0	4.0	4.0	1.0	4.0	4.0	1.0	0.0	0.0	0.0	0.0	0.0	0.0	0.0	0.0	0.0
139	PLAC	4.0	4.0	4.0	4.0	4.0	4.0	4.0	4.0	4.0	0.0	0.0	0.0	3.0	3.0	3.0	0.0	0.0	0.0
140	PLAC	4.0	4.0	4.0	4.0	4.0	4.0	4.0	4.0	4.0	0.0	0.0	0.0	0.0	0.0	0.0	0.0	0.0	0.0
141	ARL	3.0	3.0	3.0	3.0	3.0	3.0	3.0	3.0	3.0	0.0	0.0	0.0	3.0	3.0	3.0	0.0	0.0	0.0
142	PLAC	4.0	4.0	4.0	0.0	0.0	0.0	3.0	3.0	3.0	3.0	3.0	3.0	0.0	0.0	0.0	0.0	0.0	0.0
143	ARL	3.0	3.0	2.0	3.0	3.0	2.0	3.0	3.0	2.0	3.0	3.0	2.0	4.0	4.0	3.0	0.0	0.0	0.0
145	CIN	2.0	1.0	1.0	1.0	1.0	1.0	1.0	1.0	1.0	0.0	0.0	0.0	3.0	3.0	2.0	1.0	1.0	1.0
146	PLAC	1.0	1.0	1.0	0.0	0.0	0.0	0.0	0.0	0.0	0.0	0.0	0.0	0.0	0.0	0.0	0.0	0.0	0.0
147	DIM	2.0	2.0	2.0	3.0	3.0	3.0	1.0	1.0	1.0	2.0	2.0	2.0	2.0	2.0	2.0	1.0	1.0	1.0
148	DIM	4.0	3.0	0.0	3.0	3.0	0.0	3.0	2.0	0.0	0.0	0.0	0.0	3.0	2.0	0.0	2.0	2.0	0.0

149	PLAC	0.0	0.0	0.0	1.0	0.0	0.0	0.0	0.0	0.0	0.0	0.0	0.0	0.0	0.0	0.0	0.0	0.0	0.0
150	ARL	0.0	0.0	0.0	0.0	0.0	0.0	2.0	1.0	1.0	0.0	0.0	0.0	0.0	0.0	0.0	0.0	0.0	0.0
151	DIM	3.0	3.0	2.0	0.0	0.0	0.0	0.0	0.0	0.0	0.0	0.0	0.0	3.0	3.0	0.0	0.0	0.0	0.0
152	ARL	1.0	1.0	0.0	0.0	0.0	0.0	3.0	2.0	0.0	4.0	2.0	0.0	3.0	1.0	0.0	3.0	2.0	1.0
153	DIM	3.0	2.0	1.0	0.0	0.0	0.0	0.0	0.0	0.0	0.0	0.0	0.0	2.0	2.0	2.0	0.0	0.0	0.0
154	PLAC	4.0	4.0	4.0	4.0	4.0	4.0	3.0	3.0	3.0	0.0	0.0	0.0	3.0	3.0	3.0	0.0	0.0	0.0
155	PLAC	3.0	2.0	1.0	0.0	0.0	0.0	3.0	3.0	1.0	0.0	0.0	0.0	2.0	2.0	1.0	0.0	0.0	0.0
156	PLAC	3.0	3.0	3.0	3.0	3.0	3.0	3.0	3.0	3.0	1.0	1.0	1.0	2.0	2.0	2.0	0.0	0.0	0.0
157	PLAC	2.0	1.0	0.0	2.0	1.0	0.0	2.0	1.0	0.0	0.0	0.0	0.0	2.0	1.0	1.0	0.0	0.0	0.0
158	PLAC	4.0	4.0	4.0	2.0	2.0	2.0	1.0	1.0	1.0	2.0	2.0	2.0	4.0	4.0	4.0	0.0	0.0	0.0
159	PLAC	2.0	2.0	1.0	1.0	1.0	1.0	3.0	2.0	1.0	0.0	0.0	0.0	0.0	0.0	0.0	0.0	0.0	0.0
160	DIM	3.0	3.0	2.0	3.0	3.0	2.0	1.0	1.0	1.0	3.0	3.0	2.0	3.0	3.0	2.0	0.0	0.0	0.0
161-P	DIM	3.0	2.0	2.0	3.0	2.0	2.0	0.0	0.0	0.0	0.0	0.0	0.0	3.0	2.0	2.0	0.0	0.0	0.0
162-P	DIM	2.0	2.0	1.0	2.0	2.0	1.0	2.0	2.0	1.0	0.0	0.0	0.0	3.0	3.0	2.0	0.0	0.0	0.0
163-P	DIM	3.0	3.0	3.0	3.0	3.0	3.0	4.0	4.0	4.0	3.0	3.0	3.0	3.0	3.0	3.0	2.0	2.0	2.0
164-P	CIN	3.0	3.0	2.0	3.0	3.0	2.0	3.0	3.0	2.0	0.0	0.0	0.0	3.0	3.0	3.0	0.0	0.0	0.0
165-P	DIM	3.0	3.0	1.0	3.0	3.0	2.0	3.0	3.0	2.0	0.0	0.0	0.0	3.0	3.0	2.0	0.0	0.0	0.0
166-P	ARL	3.0	3.0	1.0	3.0	3.0	1.0	3.0	3.0	1.0	0.0	0.0	0.0	3.0	3.0	2.0	3.0	3.0	2.0
167-P	DIM	3.0	3.0	3.0	3.0	3.0	3.0	3.0	3.0	3.0	0.0	0.0	0.0	2.0	2.0	2.0	0.0	0.0	0.0
161-D	DIM	0.0	0.0	0.0	2.0	2.0	2.0	2.0	3.0	3.0	0.0	0.0	0.0	0.0	0.0	0.0	0.0	0.0	0.0
162-D	CIN	0.0	0.6	0.0	1.0	1.0	0.3	1.0	1.0	0.2	0.0	0.0	0.0	0.0	0.0	0.0	0.0	0.0	0.0
163-D	CIN	3.0	2.7	2.7	2.0	2.0	2.0	2.0	2.0	2.0	0.0	0.0	0.0	3.0	3.0	3.0	0.0	0.0	0.0
164-D	CIN	0.0	0.0	0.0	1.0	0.0	0.0	0.5	0.0	0.0	0.0	0.0	0.0	0.0	0.0	0.0	2.0	0.8	0.0
165-D	CIN	4.0	0.0	0.0	2.0	0.0	0.0	0.0	0.0	0.0	0.0	0.0	0.0	1.5	0.0	0.0	0.0	0.0	0.0
166-D	ARL	0.0	0.0	0.0	2.2	1.0	1.0	2.5	1.0	1.0	2.0	0.0	0.0	0.0	0.0	0.0	0.0	0.0	0.0
167-D	DIM	1.2	1.2	1.0	2.2	2.2	2.1	2.2	2.2	2.2	1.2	1.3	1.3	1.5	1.5	1.3	0.0	0.0	0.0
168	CIN	0.0	0.0	1.0	0.0	0.0	0.0	0.0	0.0	0.0	0.0	0.0	0.0	3.0	1.0	1.0	2.0	0.0	0.0
169	DIM	3.0	1.0	1.0	3.0	1.0	1.0	3.0	1.0	1.0	3.0	2.0	0.0	2.0	1.0	1.0	0.0	0.0	0.0
170	CIN	0.0	0.0	0.0	3.0	3.0	3.0	3.0	3.0	3.0	3.0	3.0	3.0	3.0	3.0	3.0	0.0	0.0	0.0
172	ARL	0.0	0.0	0.0	1.5	0.0	0.0	0.0	0.0	0.0	0.0	0.0	0.0	0.0	0.0	0.0	0.0	0.0	0.0
173	ARL	0.0	0.0	0.0	2.0	2.5	1.0	0.0	0.0	0.0	0.0	0.0	0.0	0.5	1.0	1.0	0.0	0.0	0.0
174	ARL	3.0	2.0	0.0	2.0	1.0	0.0	2.0	1.0	0.5	0.0	0.0	0.0	3.0	2.0	0.0	1.0	1.0	0.0
175	ARL	3.0	0.0	0.0	2.0	0.0	0.0	2.0	0.0	0.0	3.0	2.0	0.0	3.0	2.0	0.0	2.0	0.0	0.0
176	PLAC	0.0	0.0	0.0	3.0	2.0	2.0	3.0	2.0	2.0	2.0	1.0	0.0	3.0	2.0	2.0	1.0	1.0	1.0
177	ARL	0.0	0.0	0.0	0.0	0.0	0.0	0.0	0.0	0.0	0.0	0.0	0.0	2.0	1.0	0.0	0.0	0.0	0.0
178	ARL	3.0	1.0	0.0	3.0	1.5	0.0	2.5	1.0	0.5	3.0	2.0	0.5	2.0	1.0	0.0	2.5	2.0	0.5
179	CIN	2.0	0.0	1.0	2.0	1.0	1.0	2.0	0.0	1.0	1.5	1.5	1.0	2.0	1.0	0.5	0.0	0.0	0.0
180	CIN	1.0	1.0	1.0	0.0	0.0	1.0	2.0	1.0	1.2	1.0	0.0	0.0	0.0	0.0	0.0	0.0	0.0	0.0
181	CIN	3.0	3.0	0.0	4.0	4.0	1.0	4.0	3.0	0.0	4.0	4.0	0.0	4.0	4.0	0.0	3.0	2.0	0.0
182	CIN	0.0	0.0	0.0	1.0	2.0	1.0	1.0	2.0	0.0	3.0	3.0	3.0	2.0	3.0	0.0	0.0	1.0	0.0
183	ARL	2.5	0.0	0.0	3.0	0.0	0.0	3.0	0.0	0.0	0.0	0.0	0.0	0.0	0.0	0.0	0.0	0.0	0.0
184	DIM	0.0	0.0	0.0	0.0	0.0	0.0	0.0	0.0	0.0	0.0	0.0	0.0	1.0	1.0	1.0	0.0	0.0	0.0
185	CIN	3.0	3.0	2.0	3.0	3.0	2.0	3.0	3.0	2.0	4.0	4.0	2.0	2.0	2.0	2.0	3.0	1.0	0.0
186	DIM	4.0	4.0	4.0	1.0	1.0	1.0	1.0	1.0	1.0	0.0	0.0	0.0	3.5	3.0	2.6	0.0	0.0	0.0
187	CIN	0.0	0.0	0.0	1.0	1.0	1.0	2.0	3.0	3.0	3.0	2.0	2.0	2.0	2.0	2.0	0.0	0.0	0.0
188	CIN	3.0	1.0	0.0	2.0	2.0	2.0	1.0	1.0	1.0	2.0	2.0	0.0	2.5	1.0	0.0	2.0	1.5	1.5
189	PLAC	3.0	0.0	2.0	2.0	2.0	1.0	3.0	2.0	1.0	4.0	4.0	4.0	3.0	1.0	1.0	0.0	0.0	0.0
190	PLAC	0.0	0.0	0.0	1.0	0.0	0.0	0.0	0.0	0.0	0.0	0.0	0.0	0.0	0.0	0.0	0.0	0.0	0.0
191	PLAC	0.0	1.0	0.0	2.0	2.0	2.0	2.0	2.0	1.5	2.0	2.0	1.5	2.0	2.0	2.0	1.0	1.0	1.0
192	ARL	1.0	0.0	0.0	2.0	0.0	0.0	0.0	0.0	0.0	3.0	0.0	0.0	0.0	0.0	0.0	2.0	0.0	2.0
193	PLAC	0.0	0.0	0.0	3.0	2.0	1.0	0.0	2.0	0.0	0.0	0.0	0.0	3.0	3.0	1.0	0.0	0.0	0.0
194	ARL	2.0	0.0	1.0	3.0	0.0	1.0	3.0	2.0	1.0	0.0	0.0	0.0	4.0	0.0	1.0	3.0	0.0	0.0
195	ARL	3.0	2.0	0.0	2.0	1.0	0.0	2.0	1.0	0.0	4.0	2.0	1.0	0.0	0.0	0.0	4.0	2.0	1.0
196	DIM	2.0	2.0	2.0	0.0	0.0	0.0	2.0	2.0	2.0	3.0	3.0	3.0	2.0	2.0	2.0	2.0	0.0	0.0
197	DIM	0.0	2.0	1.4	2.0	2.0	1.5	0.0	0.0	0.0	0.0	0.0	0.0	1.5	1.5	0.5	0.0	1.0	0.0
198	DIM	0.0	0.5	0.0	0.0	0.0	0.0	2.5	3.0	0.0	2.0	2.0	2.0	1.0	1.0	0.0	1.0	1.0	0.0
199	CIN	0.0	0.0	0.0	2.0	1.0	1.0	2.0	1.0	0.0	2.0	2.0	2.0	1.5	1.5	0.7	1.5	1.5	1.5
200	PLAC	0.0	0.0	0.0	0.0	0.0	0.0	2.0	2.0	0.0	2.0	1.0	1.0	0.0	0.0	0.0	0.0	0.0	0.0
201	DIM	2.0	2.0	2.0	2.0	2.0	2.0	2.0	2.0	2.0	0.0	0.0	0.0	2.0	2.0	2.0	0.0	0.0	0.0
202	CIN	2.0	2.0	2.0	0.0	0.0	0.0	1.0	1.0	1.0	0.0	0.0	0.0	2.0	2.0	2.0	0.0	0.0	1.0
203	DIM	3.0	3.0	2.0	3.0	3.0	0.0	2.0	2.0	0.0	2.0	1.0	1.0	0.0	0.0	0.0	0.0	0.0	0.0
204	CIN	3.0	2.0	1.0	3.0	2.0	1.0	3.0	2.0	1.0	1.0	2.0	0.0	1.0	1.0	1.0	0.0	0.0	0.0
205	CIN	1.0	0.5	0.0	0.0	0.0	0.0	2.0	2.0	2.0	0.0	0.0	0.0	0.0	0.0	0.0	0.0	0.0	0.0
207	PLAC	2.0	1.5	1.0	2.0	1.5	1.0	2.0	2.0	2.0	0.0	0.0	0.0	3.0	3.0	2.5	0.0	0.0	0.0
208	PLAC	3.0	0.0	0.0	1.0	0.0	0.0	1.0	0.0	0.0	0.0	0.0	0.0	2.0	0.0	0.0	0.0	0.0	0.0
209	ARL	3.0	0.0	0.0	3.0	1.0	0.0	3.0	1.0	0.0	0.0	0.0	0.0	3.0	1.5	0.5	2.0	0.0	0.0
210	PLAC	3.0	2.0	1.0	0.0	0.0	0.0	0.0	0.0	0.0	1.0	1.0	1.0	0.0	0.0	0.0	0.0	0.0	0.0
211	ARL	3.0	3.0	3.0	2.0	2.0	2.0	1.5	1.5	1.5	0.0	0.0	0.0	3.0	3.0	0.0	0.0	0.0	0.0
212	PLAC	2.0	2.0	2.0	2.0	3.0	3.0	2.0	2.0	2.0	3.5	3.5	3.5	2.0	2.0	2.0	2.0	2.0	2.0
213	DIM	0.0	0.0	0.0	3.0	3.0	1.0	2.0	2.0	2.0	0.0	0.0	0.0	3.0	3.0	3.0	2.0	2.0	2.0
214	DIM	2.0	2.0	0.0	3.0	2.0	0.0	2.0	1.0	0.0	4.0	4.0	0.0	1.0	1.0	0.0	0.0	0.0	0.0
215	DIM	3.0	2.0	0.0	3.0	2.0	0.0	3.0	2.0	0.0	3.0	3.0	0.0	2.0	0.0	0.0	2.0	2.0	0.0
216	DIM	2.0	0.0	0.0	3.0	0.0	0.0	3.0	0.0	0.0	0.0	0.0	0.0	4.0	0.0	0.0	0.0	0.0	0.0
217	PLAC	0.0	0.0	1.0	2.0	2.0	1.0	2.0	2.0	1.0	3.0	3.0	3.0	2.0	2.0	2.0	2.0	1.0	1.0

218	DIM	1.0	1.0	1.0	2.0	2.0	2.0	3.0	3.0	3.0	2.0	2.0	2.0	4.0	4.0	4.0	4.0	4.0	3.5
219	CIN	0.0	0.0	0.0	3.0	3.0	3.0	3.0	3.0	3.0	2.0	2.0	2.0	3.0	3.0	3.0	0.0	0.0	0.0
220	DIM	1.0	0.5	0.5	3.0	3.0	2.0	3.0	3.0	1.0	1.0	1.0	0.0	1.0	1.0	0.5	1.0	1.0	0.5
221	CIN	3.0	2.0	0.0	2.0	2.0	1.0	0.0	1.0	1.0	0.0	0.0	0.0	2.0	2.0	1.0	2.0	1.0	0.0
222	DIM	3.0	3.0	3.0	3.0	3.0	3.0	3.0	3.0	3.0	2.0	2.0	2.0	2.0	2.0	2.0	0.0	0.0	0.0
223	PLAC	1.0	1.0	0.0	3.0	3.0	2.0	3.0	3.0	1.0	2.0	2.0	1.0	0.0	0.0	0.0	0.0	0.0	0.0
224	PLAC	3.0	2.0	1.0	3.0	2.0	1.0	3.0	2.0	1.0	1.0	1.0	1.0	0.0	0.0	0.0	0.0	0.0	0.0
225	PLAC	3.0	0.0	1.0	2.0	1.0	1.0	0.0	0.0	0.0	0.0	0.0	0.0	0.0	1.0	1.0	0.0	1.0	1.0
226	ARL	2.0	0.0	0.0	2.0	1.0	0.0	2.0	0.0	0.0	0.0	0.0	0.0	0.0	0.0	0.0	0.0	0.0	0.0
227	PLAC	2.5	2.5	2.0	1.0	0.5	0.5	1.0	0.5	0.5	0.0	0.0	0.0	0.0	0.0	0.0	0.0	0.0	0.0
228	ARL	0.0	0.0	0.0	2.0	2.0	0.0	2.0	2.0	0.0	0.0	0.0	0.0	2.0	1.5	0.0	0.0	0.0	0.0
229	PLAC	0.0	0.0	0.0	3.0	2.0	0.0	3.0	2.0	0.0	0.0	0.0	0.0	0.0	0.0	0.0	1.0	1.0	0.0
230	DIM	0.0	1.0	0.0	2.5	2.5	2.0	2.5	2.5	2.0	2.5	2.5	2.0	3.0	3.0	2.0	3.0	3.0	2.0
231	ARL	2.0	1.0	0.0	4.0	3.0	0.0	2.0	1.0	0.0	2.0	2.0	0.0	0.0	0.0	0.0	0.0	0.0	0.0
233	DIM	0.0	1.0	1.0	0.0	1.0	1.0	0.0	1.0	1.0	1.0	1.0	1.0	3.0	3.0	3.0	3.0	3.0	3.0
234	PLAC	2.0	2.0	1.0	2.0	1.0	0.0	3.0	2.0	0.0	2.5	3.0	1.0	3.0	2.0	1.0	0.0	1.0	1.0
235	ARL	3.0	3.0	2.0	2.0	2.0	2.0	2.0	2.0	2.0	1.0	1.0	1.0	0.0	1.0	0.0	1.0	1.0	0.0
236	CIN	2.0	2.0	1.0	0.0	0.0	0.0	0.0	0.0	0.0	0.0	0.0	0.0	2.0	2.0	0.0	0.0	0.0	0.0
237	ARL	3.0	2.0	1.0	2.0	2.0	1.0	3.0	3.0	1.0	0.0	0.0	0.0	2.0	2.0	1.0	1.0	1.0	1.0
238	ARL	1.0	0.0	0.0	2.0	1.0	0.0	0.0	0.0	0.0	3.0	1.0	2.0	2.0	1.0	2.0	0.0	0.0	0.0
239	ARL	2.0	2.0	0.0	1.0	1.0	0.0	1.0	1.0	0.0	1.0	1.0	0.0	0.0	0.0	0.0	0.0	0.0	0.0
240	PLAC	4.0	3.5	3.0	4.0	4.0	4.0	4.0	3.5	3.0	4.0	4.0	4.0	4.0	3.0	2.0	0.5	0.5	0.5

PATNO: Patient number; N = 239 (ITT population)
MEDICAT (medication): CIN = Cinnarizine 50mg, DIM = Dimenhydrinate 100mg, ARL = Arlevert (Cinnarizine 20mg + Dimenhydrinate 40mg), PLAC = Placebo
Vertigo triggering factors: Change of position (lying) (CHANGE), Bowing (BOW), Getting up (GETUP), Driving by car/train (DRIV), Head movements (inclination, twist) (HEADMOV), Eye movement (EYEMOV)
0 = baseline, 1 = after 1 week, 2 = after 4 weeks of treatment
