# Supplementary material for: The Mean Vertigo Score (MVS) Outcome Scale and Its Use in Clinical Research for Quantifying Vestibular Disorders
Source: Front Neurol. 2021 May 5;12:601749. doi: 10.3389/fneur.2021.601749 (PMC8131667; doi:10.3389/fneur.2021.601749)
Supplement: Supplementary file 3 [file Table_3.pdf]

Supplementary Table 2: Computer Output for the comparison of Arlevert and Placebo 4 weeks after onset of treatment

Sponsor : Hennig
Study : Wei&Lachin
Workfile: ARLEN.TSF
Output : CO34T2.WEL

TESTIMATE V.6.5.14
10.11.2020 - 12:04

Arlevert Study

Data Set: ITT

Wilcoxon - Test

Directional Test (stochastic ordering)
(N = 119)

Criterion	DYSTAS2	STAGGER2	ROTARY2	FALL2	LIFT2	SCOTO2	CHANGE2	BOW2	GETUP2	DRIV2	HEADMOV2	EYEMOV2
Valid No.:												
R: Placebo	58	58	58	58	58	58	58	58	58	58	58	58
T: Arlevert	61	61	61	61	61	61	61	61	61	61	61	61
Direction	T < R	T < R	T < R	T < R	T < R	T < R	T < R	T < R	T < R	T < R	T < R	T < R

Correlation	1.000000											
Matrix	0.720274	1.000000										
	0.483436	0.514723	1.000000									
	0.539784	0.641862	0.350973	1.000000								
	0.211643	0.254338	0.260148	0.286410	1.000000							
	0.231751	0.182594	0.216181	0.165951	0.244434	1.000000						
	0.605154	0.571380	0.465329	0.449367	0.206255	0.211410	1.000000					
	0.507573	0.478812	0.342901	0.380985	0.210227	0.111196	0.638009	1.000000				
	0.479332	0.404213	0.348729	0.321891	0.231021	0.188924	0.594166	0.739795	1.000000			
	0.176706	0.116189	0.062861	0.021262	0.161115	0.078995	0.140766	0.087282	0.162097	1.000000		
	0.487645	0.614576	0.280819	0.347649	0.143370	0.011850	0.516900	0.524379	0.450246	0.197422	1.000000	
	0.130071	0.137148	0.229552	0.162329	0.259039	0.072037	0.104142	0.207267	0.251342	0.132730	0.324005	1.000000

Test for Difference (two-sided, Alpha = .050)

Criterion	DYSTAS2	STAGGER2	ROTARY2	FALL2	LIFT2	SCOTO2	CHANGE2	BOW2	GETUP2	DRIV2	HEADMOV2	EYEMOV2	Directional (Stoc.Ord.)
P (two-sided)	0.0011	<.0001	<.0001	0.0123	0.0029	0.0004	<.0001	<.0001	0.0140	0.0013	0.0633	0.0339	<.0001
P(X<Y)+.5P(X=Y)	0.6607	0.6947	0.7116	0.6159	0.6136	0.6215	0.6850	0.6909	0.6183	0.6210	0.5882	0.5831	0.6420
CI-LB: 1-Alpha	0.5643	0.6015	0.6223	0.5251	0.5387	0.5541	0.5929	0.6010	0.5239	0.5473	0.4951	0.5063	0.5885
CI-UB	0.7571	0.7880	0.8008	0.7066	0.6885	0.6889	0.7771	0.7809	0.7127	0.6946	0.6813	0.6599	0.6956
